# Facioscapulohumeral dystrophy weakened sarcomeric contractility is mimicked in induced pluripotent stem cells‐derived innervated muscle fibres

**DOI:** 10.1002/jcsm.12835

**Published:** 2021-12-03

**Authors:** Camille Laberthonnière, Elva‐Maria Novoa‐del‐Toro, Mégane Delourme, Raphaël Chevalier, Natacha Broucqsault, Kilian Mazaleyrat, Nathalie Streichenberger, Véronique Manel, Rafaëlle Bernard, Emmanuelle Salort Campana, Shahram Attarian, Karine Nguyen, Jérôme D. Robin, Anais Baudot, Frédérique Magdinier

**Affiliations:** ^1^ Aix Marseille Univ, INSERM, Marseille Medical Genetics, MMG Marseille France; ^2^ Neuropathology Lyon Civil Hospices Lyon France; ^3^ Neuromyogène Institute, CNRS–UMR 5310, INSERM 1217 Claude Bernard University Lyon 1, University of Lyon Lyon France; ^4^ Reference Centre for Neuromuscular Diseases Hospital for Woman Mother Child Lyon France; ^5^ Department of Medical Genetics Timone Infant Hospital Marseille France; ^6^ Reference Centre for Neuromuscular Diseases and ALS Timone Adult Hospital Marseille France

**Keywords:** Facioscapulohumeral dystrophy, Induced pluripotent stem cells, System biology, Pathophysiology, Sarcomere, Muscle contraction, Muscle weakening

## Abstract

**Background:**

Facioscapulohumeral dystrophy (FSHD) is a late‐onset autosomal dominant form of muscular dystrophy involving specific groups of muscles with variable weakness that precedes inflammatory response, fat infiltration, and muscle atrophy. As there is currently no cure for this disease, understanding and modelling the typical muscle weakness in FSHD remains a major milestone towards deciphering the disease pathogenesis as it will pave the way to therapeutic strategies aimed at correcting the functional muscular defect in patients.

**Methods:**

To gain further insights into the specificity of the muscle alteration in this disease, we derived induced pluripotent stem cells from patients affected with Types 1 and 2 FSHD but also from patients affected with Bosma arhinia and microphthalmia. We differentiated these cells into contractile innervated muscle fibres and analysed their transcriptome by RNA Seq in comparison with cells derived from healthy donors. To uncover biological pathways altered in the disease, we applied MOGAMUN, a multi‐objective genetic algorithm that integrates multiplex complex networks of biological interactions (protein–protein interactions, co‐expression, and biological pathways) and RNA Seq expression data to identify active modules.

**Results:**

We identified 132 differentially expressed genes that are specific to FSHD cells (false discovery rate < 0.05). In FSHD, the vast majority of active modules retrieved with MOGAMUN converges towards a decreased expression of genes encoding proteins involved in sarcomere organization (*P* value 2.63e^−12^), actin cytoskeleton (*P* value 9.4e^−5^), myofibril (*P* value 2.19e^−12^), actin–myosin sliding, and calcium handling (with *P* values ranging from 7.9e^−35^ to 7.9e^−21^). Combined with *in vivo* validations and functional investigations, our data emphasize a reduction in fibre contraction (*P* value < 0.0001) indicating that the muscle weakness that is typical of FSHD clinical spectrum might be associated with dysfunction of calcium release (*P* value < 0.0001), actin–myosin interactions, motor activity, mechano‐transduction, and dysfunctional sarcomere contractility.

**Conclusions:**

Identification of biomarkers of FSHD muscle remain critical for understanding the process leading to the pathology but also for the definition of readouts to be used for drug design, outcome measures, and monitoring of therapies. The different pathways identified through a system biology approach have been largely overlooked in the disease. Overall, our work opens new perspectives in the definition of biomarkers able to define the muscle alteration but also in the development of novel strategies to improve muscle function as it provides functional parameters for active molecule screening.

## Introduction

Facioscapulohumeral dystrophy (FSHD, OMIM#158900) is a peculiar autosomal dominant form of muscular dystrophy involving predominantly specific muscles of the face, shoulder and pelvic girdles with progression to peroneal muscles,with variable weakness between muscles.^1^ FSHD usually occurs around the age of 20. The disease is considered as genetically heterogeneous but clinically homogenous with however incomplete penetrance and inter‐individual and intra‐familial heterogeneity that complicates genotype–phenotype correlation.^2^ The disease is also associated with extra muscular symptoms such as retinopathy (Coat's disease) and hearing loss.^1^

FSHD is considered as the third most common myopathy with an incidence of 1/20 000.^3^ The disease is linked to the deletion of an integral number of repetitive macrosatellite elements (D4Z4) in the subtelomeric 4q35 region.^4^ This deletion occurs on A‐type alleles that define the region distal to the last D4Z4 repeat.^5^ Healthy individuals carry more than 11 copies of D4Z4 while this number drops below a threshold of 10 repetitive units (RUs) in patients.^4^ This category is the most frequent (Type 1, FSHD1, 95%). In a smaller proportion of cases (Type 2, FSHD2, approximately 5%), the D4Z4 array is not shortened, but 80% of these patients carry a variant in the gene encoding the SMCHD1 (structural maintenance of chromosomes flexible hinge domain containing 1) chromatin‐associated factor.^6^

The current and most prevalent molecular model proposed as causative of FSHD associates reduction in the number of D4Z4 repeats (FSHD1) or loss of SMCHD1 function (FSHD2) to hypomethylation of the CG‐rich D4Z4 sequence and transcription of the most distal copy of the *DUX4* gene through the last D4Z4 unit and abutting telomeric sequence.^7^ In FSHD, DUX4 is detectable in muscles and non‐muscular tissues.^8^ DUX4 is also expressed in normal adult muscle^8^, ^9^ albeit at a lower level compared with FSHD samples where it is expressed in 1/1000 nuclei in myotubes.^9^

DUX4 regulates a number of genes, among which many are involved in immune response and inflammation.^10^ Some of them, such as *MBD3L2*, *TRIM43*, *LEUTX*, *ZSCAN4*, or *KHDC1L* are considered as robustly up regulated in the disease. However, their functions in muscle biology remain largely unknown^10^ and the muscle defect leading to FSHD and what triggers the typical muscle weakness poorly defined. Consequently, only a few markers are currently used for drug screening and development of therapies.

*SMCHD1* is mutated in another unrelated rare developmental syndrome, Bosma arhinia and microphthalmia (BAMS) likely associated with defects in neural crest cells migration.^11^, ^12^ BAMS patients also display D4Z4 hypomethylation and *DUX4* expression^12^, ^13^ despite the absence of muscle phenotype in these patients, indicating that alternative genes/pathways are implicated in the two diseases.

To identify FSHD‐specific pathways and understand muscle alterations that are specific to FSHD cells, we used induced pluripotent stem cells differentiated into innervated muscle fibres.^14^ We compared the transcriptome of FSHD‐derived cells with cells from healthy donors but also cells from patients affected with BAMS by RNA Sequencing using classical pipelines but also applied MOGAMUN, a recently developed algorithm able to find active modules from multiplex biological networks by integrating protein–protein interactions, biological pathways and co‐expression.^15^ Interesting areas are located automatically, by finding highly deregulated and dense subnetworks. Importantly, MOGAMUN also considers genes that are not significantly differentially expressed, but which can be key to connect highly deregulated genes.

We found changes in the expression level of several sarcomeric proteins as a hallmark of FSHD cells and confirmed these findings *in vivo* in biopsies and using functional assays. Our results emphasize the importance of system biology approaches for inferring the transcriptional landscape of complex diseases. This work also provides reliable readouts for monitoring FSHD muscle function, including those that might be secondary or downstream of DUX4, opening new grounds for the identification of therapeutic targets able to correct the typical muscle weakening that precedes muscle atrophy and fatigue.

## Materials and methods

### Samples

All individuals have provided written informed consent for the collection of samples and use for research. The study was performed in accordance with the Declaration of Helsinki. Controls are randomly chosen individuals selected in the same age range and sex representation as patients. Samples are listed in *Supporting information Table* S1 and described in Dion *et al*.^13^ For validation of RNA‐Seq data, biopsies from adult patients were obtained from affected patients diagnosed by expert neurologists.

### Neuromuscular differentiation

The hIPSc were differentiated into functional muscles according to the protocol described in Mazaleyrat *et al*.^14^ Cells were collected 30 days post‐differentiation.

### RNA extraction, quality control, and library preparation

Total RNA was extracted using the RNAeasy kit (Qiagen) following the manufacturer's instructions. Quality, quantification, and sizing of total RNA were evaluated using the RNA 6000 Pico assay (Agilent Technologies Ref. 5067‐1513). Only samples with a RNA integrity number (RIN) >9 were further used. RNA‐Seq libraries were generated from 600 ng of total RNA using TruSeq Stranded mRNA Library Prep Kit and TruSeq RNA Single Indexes kits A and B (Illumina, San Diego, CA, USA), according to the manufacturer's instructions. Libraries were sequenced at the IGBMC GenomEast facility using a HiSeq 4000 1x50bp.

### RNA‐Seq data processing and differential expression analysis

We assessed fastq sequence data quality using FastQC v0.11.5 and trimmed the reads to remove adapter sequences and low‐quality bases using DimerRemover v0.9.2. The resulting trimmed single‐end reads were aligned using STAR v2.5.3a^16^ to the GRCh38 human genome release. BAM files were indexed using Sambamba (v0.6.6) after ordering them by coordinates. Differentially expressed genes (DEGs) were extracted at the fold‐change cutoff ≥2 and an FDR corrected P value of 0.05 (named after FDR) in DESeq2 analysis. The numbers of transcripts per million (TPM) for each sample are shown in *Data* S1. RNA‐Seq data were analysed using MOGAMUN, a multi‐objective genetic algorithm that identifies active modules (i.e. highly interconnected subnetworks with an overall deregulation) in a multiplex biological network composed of different layers, where each of them represents physical and/or functional interactions.^15^ We used as input for MOGAMUN the resulting FDR‐corrected p‐values, and a multiplex biological network composed of three layers or undirected interactions from.^17^ The first layer corresponds to physical protein‐protein interactions obtained by merging the CCSB Interactome database^18^ and several databases from the PSICQUIC portal.^19^ The second layer contains data of pathways, obtained using the R‐package *graphite*. The third layer corresponds to absolute Spearman correlation superior or equal to 70%, calculated using the RNA‐Seq expression data of 32 tissues and 45 different cell lines.^20^ We ran MOGAMUN 30 times with the default parameters. The resulting accumulated Pareto front is composed of active modules, which contain different number of genes, including significant ones considering an absolute log fold change >1 and FDR <0.05. The raw RNA‐Seq data and raw count matrix were deposited at the NCBI Gene Expression Omnibus (https://www‐ncbi‐nlm‐nih‐gov.gate2.inist.fr/geo/) under the accession number GSE169724 accessible using the following secure token yjmnmwkmnjajbcv.

### Quantitative RT‐PCR

Reverse transcription of 1 μg of total RNA was performed using the Superscript IV First‐Strand cDNA Synthesis kit (Life Technologies) with a mix of oligo dT and random hexamers. Primers are listed in (*Table* S2). Real‐time PCR amplification was performed on a LightCycler 480 (Roche) using the SYBR green master mix. Quantitative PCR comply with the MIQE guidelines.

### Imaging and analysis of calcium transients

Fluorescent Ca2+ indicator FLUO8‐AM (5‐μM Fluo8‐AM and 0.04% Pluronic acid, AAT Bioquest) was added to cell culture medium. After 30 min incubation at 37°C, imaging was performed on a Fast‐Imaging Observer (Zeiss). For calcium handling measurements, fluorescence was excited at 488 nm and emission collected at >509 nm. Images were acquired as a time series. Different regions were tracked to record the fluorescent intensity at a 10× magnification during 30 s and an interval of 200.0 ms corresponding to 151 frames. The amplitude of the signal was automatically determined using the Zen software (Zeiss) and reported for individual cells. For measurement of fibre contraction, images were processed with IMARIS (Bitplane) using the automatic spots tool. The length of fibre contraction was determined as the shifting over time of randomly distributed points (*n* = 1200 data points per condition) along independent muscle fibres (*y*‐axis).

## Results

### HiPSC‐derived myotubes express low levels of DUX4 and DUX4 target genes

**Figure 1:**
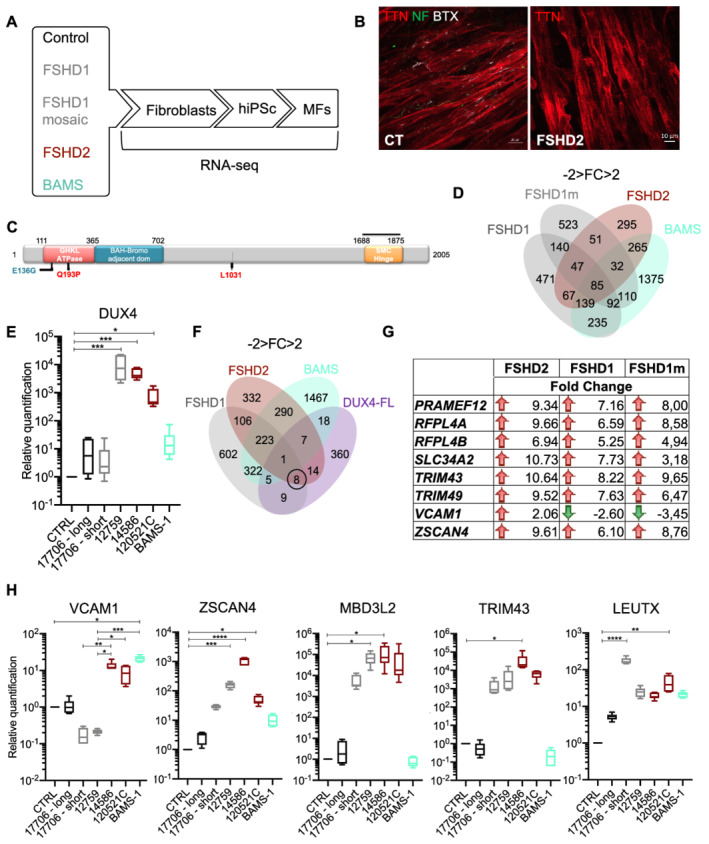
Bosma arhinia and microphthalmia (BAMS) and facioscapulohumeral dystrophy (FSHD) patient's cells progenies express the pathogenic *DUX4‐fl* transcript and DUX4 target genes. *(A)* Schematic overview of the samples used and steps of analysis. Induced pluripotent stem cells (hiPSCs) were derived from primary fibroblasts from healthy donors, patients affected with Type 1 FSHD (one FSHD1 patient carrying 7 D4Z4 repeated units (12759) and one patient with mosaicism [25% of the cells carry a 4qA allele with 2 D4Z4 repeated units; 75% of cells carry a 4qA allele with 15 D4Z4 repeated units (17706)], FSHD2 (14586; *c*.573A>C; *p*.Q193P), BAMS (BAMS‐1; c.407A>G, *p*.E136G) (*Table* S1).^13^ Fibroblasts were reprogrammed into human induced pluripotent stem cells (hiPSCs), for FSHD1 mosaic cells, hiPSCs clones carrying the contracted D4Z4 or the healthy D4Z4 allele were isolated separately. Cells were described in Dion *et al*.^13^ All hiPSCs clones were differentiated into functional muscle fibres (MFs). Gene expression analysis was performed by high‐throughput RNA‐sequencing. For each condition, two biological replicates corresponding to two independent differentiation experiments were used. DEGs were selected based on a minimum two‐fold change and statistical significance of FDR, compared with control cells. In complement to classical RNA‐Seq pipeline analysis, we applied MOGAMUN, a recently developed algorithm aimed at revealing active modules in multiplex biological networks. *(B)* Representative Z stacks of muscle fibres stained for Titin (TTN, red), neurofilament (NF, green), and acetylcholine receptors (AChR) using alpha bungarotoxin coupled with Alexa 555 (White) in control cells or FSHD2 cells at Day 30 post‐differentiation. *(C)* Schematic representation of the SMCHD1 protein and position of mutations in BAMS (cyan) or FSHD2 patients (red). BAMS‐1 (E136G) carries a missense mutation in the ATPase domain reported as a gain of function. FSHD2 patient #14586 carries a mutation in the ATPase domain (Q193P) reported as a loss of function in the ATPase activity. FSHD2 samples #12051C carrying a synonym mutation at position p.1031 was used as additional control for validation. *(D)* Venn diagrams for comparison of genes that are differentially expressed in FSHD1, FSHD2 and BAMS MFs compared with controls with a fold‐change −2 < FC > 2 and an FDR < 0.05 in. *(E)* Analysis of *DUX4* expression in the different samples by RT‐qPCR. *(F)* By comparing our list of DEGs in FSHD1, FSHD2, or BAMS vs. control MFs with DUX4 target genes, only 1 gene, *OAS2* (encoding the 2′‐5′‐oligo adenylate synthetase 2), is common between FSHD1, FSHD2, and BAMS MFs, 9 DUX4 target genes are specifically deregulated in FSHD1 MFs, 14 in FSHD2 MFs and 18 in BAMS MFs; 8 are common to FSHD1 and FSHD2. DUX4 target genes that are differentially expressed are listed on the right of the Venn diagram. *(G)*. DUX4 target genes that are differentially expressed in FSHD1, FSHD1 mosaic, and FSHD2 MFs. Red arrows correspond to genes that are up‐regulated and green arrows, to down‐regulated genes. The fold change is indicated for all of them. *(H)* Validation by RT‐qPCR of *DUX4* and selected DUX4 target genes, *ZSCAN4* (zinc finger and SCAN domain containing protein 4), *MBD3L2* (methyl CpG binding domain protein 3 L2), *TRIM43* (tripartite motif containing 43), *LEUTX* (leucine twenty homeobox), *VCAM1* (vascular cell adhesion molecule 1) expression in MFs. Box plots display the results of biological and technical triplicates for each group of samples [controls, FSHD1 short corresponds to the clone containing the contracted D4Z4 allele for the mosaic patient (17706) and FSHD1 long corresponds to its isogenic control; FSHD1 (12759) FSHD2 (14586; 120521C) and BAMS‐1]. Statistical significance was determined using a Kruskal–Wallis statistical test. *P value <0.05, **P value <0.005, ***P value <0.0005, and ****P value <0.00005.

In order to identify pathways involved in FSHD which are specific to the muscle, we took advantage of our collection of induced pluripotent stem cells^13^ and our procedure for production of innervated and contractile skeletal Muscle Fibres (MFs)^14^ to analyse gene expression in cells from FSHD1 or FSHD2 patients compared to healthy donors (*Figure* 1A,B). Given the implication of SMCHD1 in both FSHD2 and BAMS, but the absence of muscle symptoms in BAMS, RNA Seq was also carried in BAMS MFs to select DEGs that are specific to FSHD. For FSHD1, RNA Seq was performed in cells from one patient affected with FSHD carrying a short D4Z4 allele (#12759; 7 RU) and cells from a mosaic patient (#17706; 25% of mosaicism, clinically affected). For this patient, we compared two isogenic clones, one with a short allele (2 RU, 4qA haplotype, diseased clone) and one with a long healthy allele (15 RU; 4qA haplotype).^13^ For patients with *SMCHD1* variants, we compared FSHD2 cells with a missense mutation in the ATPase domain, never reported in BAMS that abrogates SMCHD1 enzymatic activity (#14586; Q194P)^13^ to BAMS cells with a variant in the ATPase domain (BAMS‐1; E136G) that causes a gain of the enzymatic activity^12^ (*Figure* 1C).

Differential expression analysis in FSHD1, FSHD1 mosaic, FSHD2, and BAMS MFs revealed 471 transcripts dysregulated in FSHD1 MFs, 523 in FSHD1 mosaic compared to its isogenic control, 295 are specific to FSHD2 and 1375 genes only dysregulated in BAMS MFs (−2 > fold change >2; FDR < 0.05) (*Figure* 1D). Among them, 140 DEGs are shared between FSHD1 and FSHD1 mosaic MFs, 67 are common between FSHD1 and FSHD2 MFs, 265 between FSHD2 and BAMS MFs, and 235 between BAMS and FSHD1 MFs. A total of 85 dysregulated transcripts are common between the four types of samples.

As reported elsewhere,^21^, ^22^ *DUX4* mRNA is not detectable by RNA Seq in any of the samples but could be detected by classical RT‐PCR methods (*Figure* 1E). *DUX4* expression is significantly increased in FSHD1 (12759) and FSHD2 (14586 and 12052C) MFs but expressed at a low level in FSHD1 mosaic cells carrying the short allele or BAMS MFs (*Figure* 1E).

DUX4 target genes are considered as more robust markers of FSHD muscle. We compared FSHD1, FSHD2, and BAMS DEGs with the list of DUX4 targets.^10^ Eighteen of these genes are dysregulated in BAMS cells but only 8 are specific to FSHD1 and FSHD2 MFs (*Figure* 1F) including *TRIM43* (FSHD1: FDR 2.87E^−06^, FSHD1 mosaic: FDR 1.10e^−07^, FSHD2: FDR 9.02e^− 11^) and *ZSCAN4* (FSHD1: FDR 0.042, FSHD1 mosaic: FDR 4,16e^−07^, FSHD2: FDR 1.780e^−08^) (*Figure* 1G). Among them, *VCAM1* expressed by adipogenic progenitors (FAPs), required for satellite cells niche regulation or inflammatory response^23^ and induced upon DUX4‐FL overexpression^10^ is down‐regulated in FSHD1 MFs (FSHD1: FDR 2.13e^−07^) and FSHD1 mosaic MFs [false discovery rate (FDR) 5.43e^−29^] but up‐regulated in FSHD2 MFs (FDR 1.01e^−08^) (*Figure* 1G). These data were confirmed by RT‐qPCR as *VCAM1* is down‐regulated only in FSHD1 MFs compared to control cells but up‐regulated in FSHD2 and BAMS cells (*Figure* 1H). By RT‐qPCR, we confirmed the up‐regulation of *ZSCAN4* and *MDB3L2* in FSHD1 (12759) and FSHD2 (14586) samples. *LEUTX* expression is significantly increased in FSHD1, FSHD2, and BAMS MFs. *TRIM43* is up‐regulated in one FSHD2 MF sample only (14586). Altogether, these results indicate that FSHD1, FSHD2, or BAMS patient's MFs cannot be distinguished based on *DUX4* expression or on the differential expression of DUX4 targets.

### Expression analysis of hiPSCs‐derived functional muscle fibres highlights pathways related to sarcomere structure and muscle contraction in FSHD

**Figure 2:**
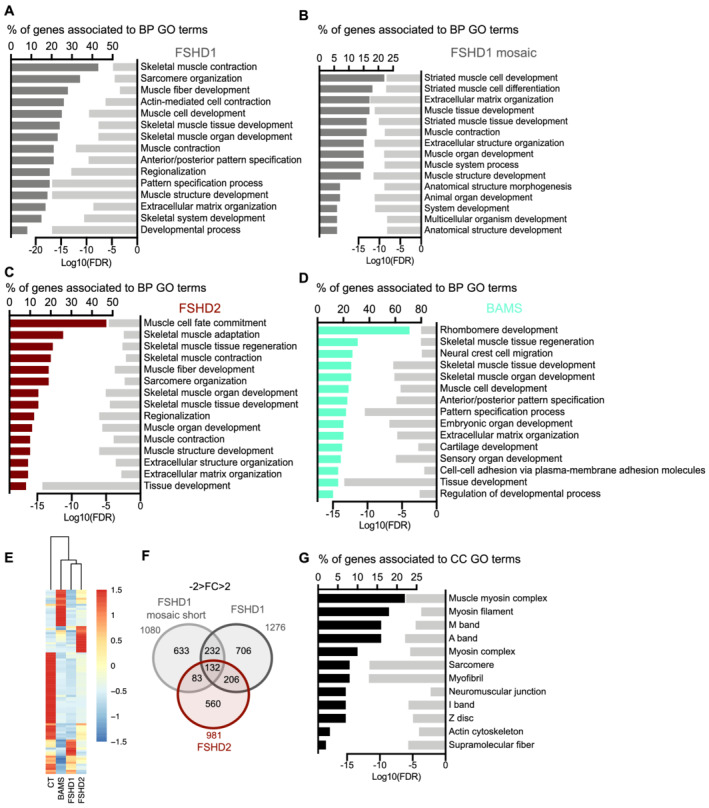
Fi Differentiation of hiPSCs from FSHD1, FSHD1 mosaic, FSHD2, and Bosma arhinia and microphthalmia (BAMS) into functional muscle fibres revealed defects in sarcomere‐related functions in cells affected by FSHD. Overrepresentation test analyses were performed using enrichGO from the R package clusterProfiler (v3.10.15). We identified biological processes (BP) with a false discovery rate (FDR) <0.05. *(A)*. Biological pathways (BPs) corresponding to enrichment analysis of DEGs in FSHD1 vs. control MFs filtered on −2 < FC > 2 and FDR < 0.05. Bar plot in the left represents the percentage of genes that are deregulated and associated with a GO‐term shown in the right column. Light grey bars in the right represent the enrichment score (Log10 of FDR) for each GO‐term. *(B)* BP corresponding to enrichment analysis of DEGs in FSHD1 mosaic vs. its isogenic control MFs filtered on −2 < FC > 2 and FDR < 0.05. Bar plot in the left represents the percentage of genes that are deregulated and associated with a GO‐term shown in the right column. Light grey bars in the right represent the enrichment score (Log10 of FDR) for each GO‐term. *(C)* BP corresponding to enrichment analysis of DEGs in FSHD2 vs. control MFs filtered on −2 < FC > 2 and FDR < 0.05. Bar plot in the left represents the percentage of genes that are deregulated and associated with a GO‐term shown in the right column. Light grey bars in the right represent the enrichment score (Log10 of FDR) for each GO‐term. *(D)* BP corresponding to enrichment analysis of DEGs in BAMS vs. control MFs filtered on −2 < FC > 2 and FDR < 0.05. Bar plot in the left represents the percentage of genes that are deregulated and associated with a GO‐term shown in the right column. Light grey bars in the right represent the enrichment score (Log10 of false discovery rate) for each GO‐term. *(E)* Heatmap for genes associated with muscle GO terms with dendrogram branches associating the different groups of samples based on transcripts per million (TPM) values. Distance was determined by Manhattan and Clustering, Ward.D2. *(F)* Venn diagram for FSHD MFs. FSHD1 and FSHD2 DEGs were obtained by comparisons with control MFs, FSHD1 mosaic (contracted D4Z4 allele only). By comparison to the clone with the normal D4Z4 allele (15 RUs), a list of 132 DEGs common to the three conditions was obtained. *(G)* Cellular components analysis for the list of 132 DEGs in common between FSHD1, FSHD1 mosaic, and FSHD2 MFs. Genes with the higher FC are related to muscle contraction. Twenty genes belong to ‘supramolecular fibres’ cellular component (*P* value, 2.10e^−06^), 17 genes to ‘myofibril’ (*P* value, 2.19e^−12^), 16 genes to ‘sarcomere’ (*P* value, 2.63e^−12^) and 12 to ‘actin cytoskeleton’ (*P* value, 9.4e^−05^).

In FSHD1, FSHD1 mosaic and FSHD2 MFs, out of the 15 top biological pathways (BPs) that are dysregulated mainly concern muscle organization and functions with GO terms related to ‘sarcomere organization’, ‘skeletal muscle contraction’, and ‘muscle structure development’ in all three categories of FSHD MFs (10/15; 8/15; 12/15, respectively. *Figure* 2A–C). BPs related to muscle function are less enriched in BAMS MFs (4 out of 15) with a significant dysregulation of BP relevant to the disease phenotype such as ‘neural crest cell migration’ and ‘cartilage development’ together with developmental processes such as ‘Rhombomere development’, ‘anterio/posterior pattern specification’, ‘embryonic organ development’, ‘sensory organ development’, and ‘regulation of developmental process’ (*Figure* 2D). In all four conditions, GO term corresponding to ‘extracellular matrix organization’ is represented (*Figure* 2A–D) suggesting common defects.

The 132 DEGs restricted to FSHD samples are all associated with sarcomeric function (*Figure* 2E,F). Associated cellular components highlighted the dysregulation of muscle ultrastructure, sarcomere organization and neuromuscular junction (*Figure* 2G) with 20 genes that belong to ‘supramolecular fibres’ cellular component, 17 genes to ‘myofibril’, 16 genes to ‘sarcomere’ and 12 to ‘Actin cytoskeleton’.

### Active module analysis reveals perturbed sarcomeric organization in FSHD

To further identify subnetworks of interest and thus reveal pathways associated to the disease, we analysed our RNA‐Seq data using MOGAMUN, a multi‐objective genetic algorithm that integrates expression data into multiplex biological networks.^15^ These multiplex network are composed of three layers, which correspond to protein–protein interactions (PPI, blue edges), BPs (red edges), and an absolute correlation of expression (referred to as co‐ expression, yellow edges) of at least 70%, calculated using the RNA‐Seq expression data of 32 tissues and 45 different cell lines. Comparison between the pathological and control conditions revealed between 16 and 23 subnetworks considered as active modules. Based on the redundancy of genes in these different networks, we further selected the most similar active modules for in‐depth analysis.

**Figure 3:**
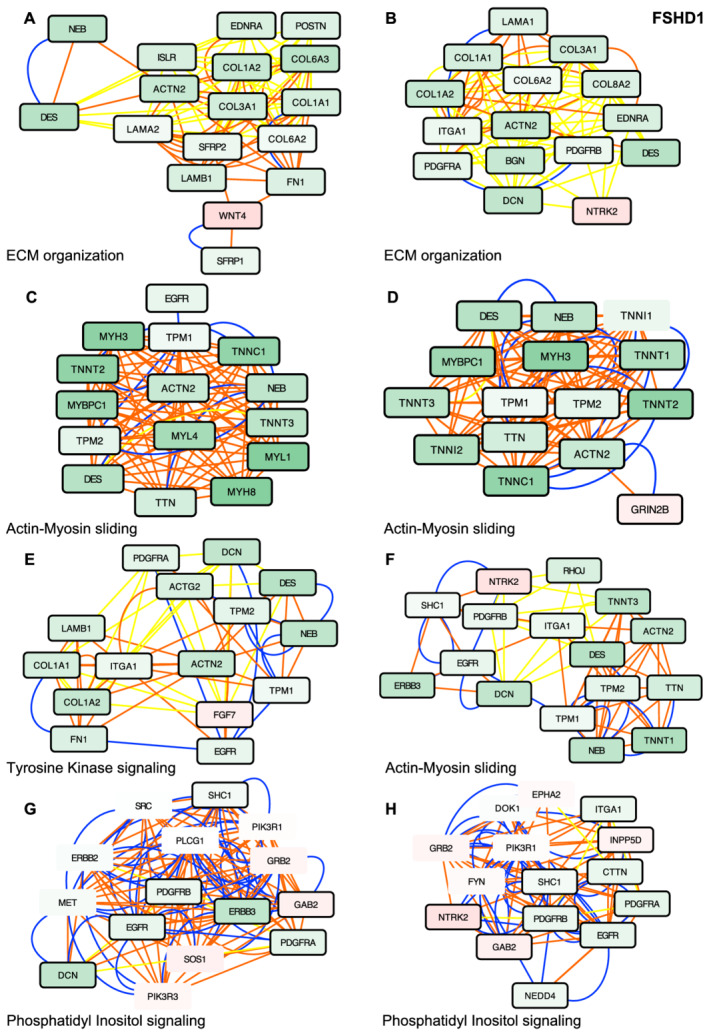
Active modules analysis in muscle fibre (MF) derived from FSHD1 hiPSCs reveals defects in sarcomeric protein network. *(A–H)* Representative active modules sampled out of 23 nodes for FSHD1 datasets using the MOGAMUN algorithm. Integration of protein–protein interactions (blue lines), biological pathways (orange lines), and co‐expression data (yellow lines) with our lists of differentially expressed genes (DEGs) enabled the identification of active modules for each category of samples. Up‐regulated nodes are coloured in red, and down‐regulated ones are in green. The intensity of the colour reflects the fold change. Thickness of the dark line around each rectangle reflects the level of significance [false discovery rate (FDR) <0.05 and 1 < FC > 1]. Each active module contains between 15 and 16 genes. Genes corresponding to each nodes were analysed using g:Profiler to define corresponding molecular function and *P* value. *(A)* Extracellular matrix (ECM) organization, *P* value 7.5e^−9^. *(B)* Extracellular matrix organization, *P* value 1.1e^−9^. *(C)* Actin–myosin filament sliding, *P* value 9.8e^−24^. *(D)* Actin–myosin filament sliding, *P* value 7.9e^−35^. *(E)* Transmembrane receptor tyrosine kinase signalling, *P* value 3.3e^−18^. *(F)* Actin–myosin filament sliding, *P* value 7e^−9^. *(G)* Phosphatidyl inositol‐mediated signalling, *P* value 2.13e^−14^. *(H)* Phosphatidyl inositol‐mediated signalling, *P* value 1. 3e^−14^.

Consistent with BP enriched in FSHD, several active modules retrieved by MOGAMUN in FSHD1 links the down‐regulation of factors involved in the organization of the sarcomere, thin filament, and contractile activity or component of the extracellular matrix (ECM) (*Figures* 3A,B and S2A–C). A majority of these active modules highlight the down‐regulation of *ACTN2* encoding the muscle specific actinin alpha 2 cytoskeletal proteins localized to the sarcomeric Z‐disc where it contributes to the anchoring of the myofibrillar actin filaments. In these different active modules, *ACTN2* is connected (pathways and protein‐protein interactions) to genes encoding ECM factors involved in cell adhesion, migration or response to mechanical stimulus and tensile strength such as *BGN*, *DCN*, *POSTN*, *LAMC3*, *FN1*, *ITGA4*, *COL1A1*, *COL1A2*, *COL3A1*, and *COL6A*3 but also to genes encoding proteins involved in sarcomeric organization and muscle contraction (*TNNT1*, *TNNT2*, *TTN*, *MYBPC1*, *TNNC1*, *DES*, and *NEB*) (*Figures* 3C–F and S2D–G). The redundancy of these active modules reveals pathways that might trigger the typical muscle weakening defined as a clinical hallmark of the disease.

Another noticeable active module is driven by the up‐regulation of *WNT4* (*Figure* 3A), required for dorsal to ventral patterning, motor neurons connectivity during embryogenesis and control of the mechanical properties and quiescent state of muscle stem cells. *WNT4* up‐regulation is associated with down‐regulation of its antagonists, *SFRP1* (secreted frizzled‐related protein 1), *SFRP2* (secreted frizzled‐related protein 2) that inhibit WNT signalling (*Figure* 3A) and down‐regulation of *ISLR* that activates WNT signalling and regulates skeletal muscle regeneration.^24^ Changes in the canonical WNT β‐catenin signalling pathway that are specific to FSHD cells further highlight potential developmental defects or defects in muscle regeneration process.

An additional active module retrieved by MOGAMUM associates down‐regulation of sarcomeric proteins to up‐regulation of *GRIN2B* (glutamate ionotropic receptor NMDA type Subunit 2B), also expressed in muscle and encoding a subunit of the NMDA receptor ion channel, which links excitatory synaptic transmission and calcium permeability (*Figure* 3D). Other active modules correspond to genes involved in phosphatidyl inositol‐mediated signalling (*Figures* 3G,H and S3A–G) and associates membrane receptors such as EGFR, ERBB3, and ERBB4, expressed in muscle progenitors and involved in regulating the balance of myogenic cell fate to signalling cascades and ECM or sarcomeric components, through co expression (yellow edges), pathways (red edges) or PPi (blue edges) nodes suggesting a decreased in proliferative progenitor cells.

**Figure 4:**
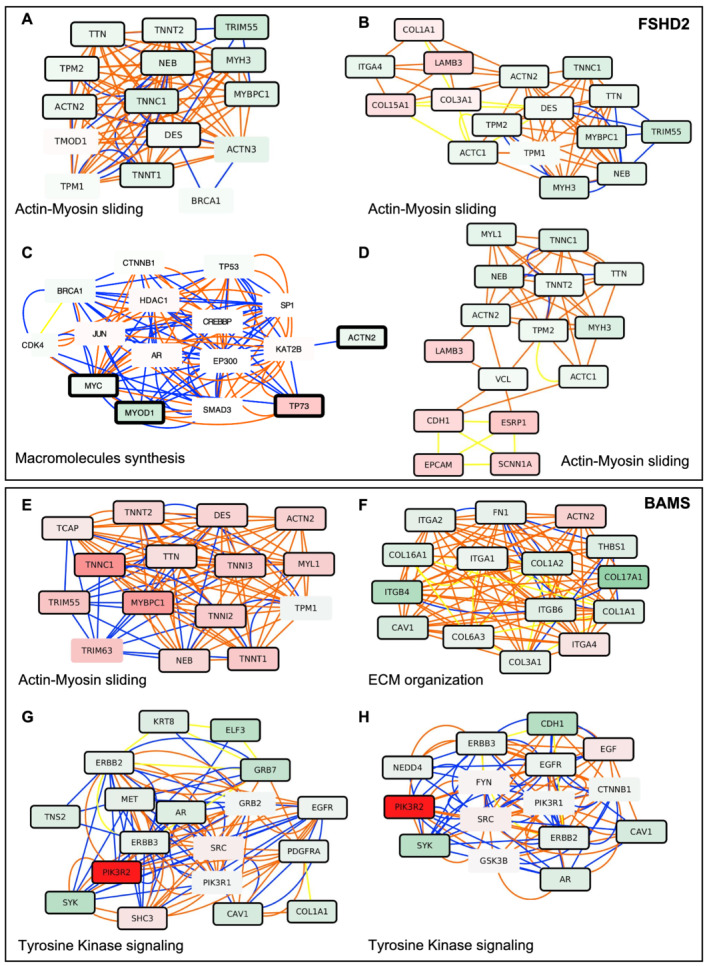
Network‐based analysis of hiPSCs derived into functional muscle fibres highlights active modules that are specific to each pathology. Representative active modules sampled from the accumulated Pareto front of 30 runs for FSHD2 datasets using the MOGAMUN algorithm. The colour of the nodes represents the fold change, where green and red nodes correspond to down‐regulated and up‐regulated genes, respectively. Nodes with bold black border correspond to genes significantly differentially expressed (FDR < 0.05 and −1 > FC > 1). The colour of the edges corresponds to the layer of the multiplex where the interaction comes from. Blue for protein–protein interactions, orange for biological pathways, and yellow for co‐expression. Each active module contains between 15 and 18 genes. Genes corresponding to each nodes were analysed using g:Profiler to define corresponding molecular function and *P* value. *(A–D)* Selection of representative active modules for FSHD2 cells. *(A)* Actin–myosin filament sliding, *P* value 2.3e^−28^. *(B)* Actin–myosin filament sliding, *P* value 7.5e^−21^. *(C)* Positive regulation of macromolecules synthesis, *P* value 4.2e^−11^. *(D)* Actin–myosin filament sliding, *P* value 1.04e^−15^. *(E–H)* Selection of representative active modules for BAMS cells. *(E)* Actin–myosin filament sliding, *P* value 2.3e^−28^. *(F)* Extracellular matrix organization, *P* value 8.5e^−15^. *(G)* Transmembrane receptor tyrosine kinase signalling, *P* value 5.1e^−14^; ERBB2 signalling, *P* value 1.85e^−13^. *(H)* Transmembrane receptor tyrosine kinase signalling, *P* value 1.85e^−14^; ERBB2 signalling, *P* value 8.5e^−13^.

In FSHD2 MFs, MOGAMUN retrieved several active modules that are similar to the ones obtained in FSHD1 cells (*Figure* 4A–D) with dysregulation of genes encoding factors involved in ECM, sarcomere organization, and muscle contraction, including *MURF2* (*TRIM55*; FSHD1: FDR 2.81e^−09^; FSHD1 mosaic: FDR 6.49e^−06^; FSHD2: FDR 7.60e^−17^; BAMS: FDR 1.09e^−13^), implicated in sarcomere formation, myogenic differentiation and required for adaptation of striated muscle to stress but also *ACTN2*, *TTN*, *TNNT*, or *TNNC* genes. In 4 out of 17 modules involving genes implicated in the ‘regulation of transcription’ (*Figures* 4C and S5E), *TP73* that controls the early stage of myogenic differentiation is up‐regulated while *MYOD* is decreased, revealing as in FSHD1 cells, a lower expression of factors required for muscle differentiation or functioning of the contractile apparatus, strengthening previous observations.

In BAMS, we noticed a strong up‐regulation of *PIK3R2* (Phosphoinositide‐3‐Kinase Regulatory Subunit 2) required for cell growth, survival, proliferation and motility, and enrichment in nodes associated with transmembrane receptor tyrosine kinase signalling (*Figures* 4G,H and S7A–F). Most of the other active modules involve genes encoding ECM proteins with a down‐regulation of FILAMIN, FIBRONECTIN, different INTEGRIN, LAMININ, and COLLAGEN isoforms suggesting defects in ECM organization and cell communication/migration or adhesion (*Figure*s 4E,F and S6A–E). However, we did not observe any significant decrease in expression of genes encoding sarcomeric proteins as in FSHD (*Figures* 3 and 4) further arguing in favour of the high relevance of muscle‐related modules retrieved in all FSHD samples.

In all conditions, a number of pathways associate proteins involved in DNA replication (*Figure* S9A–H) with an overall decrease of a few genes required for replication initiation such as *CDC6* (*Figure* S9C–H), MCM10 (*Figure* S9C–F), or *MCM3*; *4*; *6* (*Figure* S9G,H), which might illustrate in this post‐mitotic cell model, a decrease in proliferative progenitor cells as also illustrated by the decreased expression of muscle progenitor cell surface markers such as *ERBB3* or *PDGFRA*.

Interestingly, both in FSHD1 and FSHD2 but not in BAMS, we observed a decreased expression of several genes such as *ACTA1*, *ACTN2*, *TNNC1*, *TNNT2*, *TPM1*, or *TCAP* that have been associated with hearing impairment and a decreased perception of sounds, one of the extra‐muscular symptoms reported in many FSHD patients.

By applying RNA Seq and multiplex network analyses on biological replicates of hiPSC‐derived innervated muscle fibres from different donors, we identified defects in the sarcomeric apparatus that is specific to FSHD1 and FSHD2 also relevant to the phenotype of patients, including extra muscular symptoms opening novel perspective for understanding but also correcting the muscle phenotype.

### In FSHD, alteration of the contractile apparatus is consistently observed in all hiPSC‐derived contractile muscle fibres

To confirm RNA‐Seq data, we further analysed the expression of different genes by RT‐qPCR in cells used for the RNA Seq but also additional hiPSC‐derived innervated muscle fibres (*Table* S1; *Figure* S10). In hiPSC‐derived MFs, we confirmed the significant down‐regulation of *ACTN2* (FSHD1, FSHD1 mosaic), *TRIM55* (FSHD2), *TPM1* (FSHD1; FSHD1 mosaic), *MyBPC1* (FSHD1), *NEB* (FSHD1; FSHD1 mosaic; FSHD2), *TNNC1* (FSHD1, FSHD1 mosaic), and *TNNI2* (FSHD1) together with the up‐regulation of *ACTA1* (FSHD1 mosaic). By analysing the expression profile in fetal FSHD1 biopsies and adult biopsies from FSHD1 and FSHD2 patients (*Table* S1), we observed similar variations in expression for *ACTA1*, *COL1A2*, *TPM1*, *NEB*, or *TNNC1*, with however, some variations between samples likely explained by the heterogeneity in the type of muscle (deltoid/quadriceps), age at biopsy, or level of damage (*Figure*s 5A and S10).

**Figure 5:**
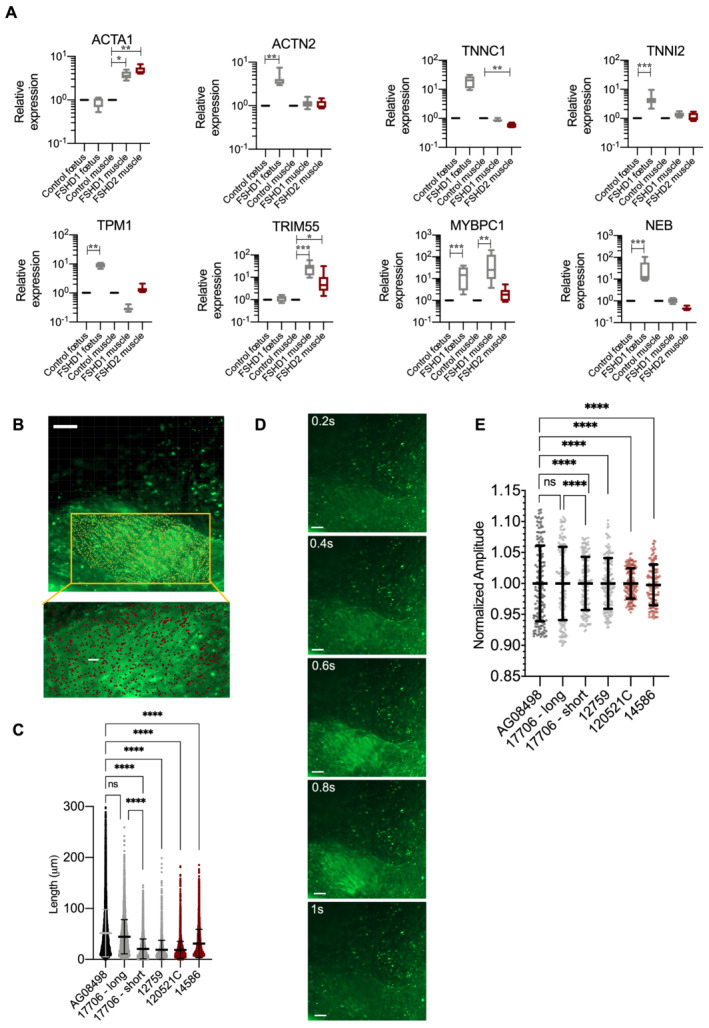
Sarcomeric dysfunction and calcium release *in vivo* and innervated muscle fibres derived from patients affected with FSHD. *(A)* RT‐qPCR was performed in technical triplicates for each biopsy sample. For each group, two different biopsies from two different individuals were used (*Table* S1). Expression was normalized to three housekeeping genes (*GAPDH*, *HPRT*, and *PPIA*). Statistical significance was determined by Kruskal–Wallis statistical test. *P value <0.05, **P value<0.005, ***P value <0.0005, and ****P value<0.00005. *(B)* Muscle contractions were recorded for 30 s with images taken every 200 ms in live innervated muscle fibres (*Movie* S1). *(C)* Scattergram displays the length of contraction determined as the shifting over time of randomly distributed point (*n* = 1200 data points per condition) along independent muscle fibres (*y*‐axis) analysed using the Imaris software in the different conditions. Data were analysed using a Brown–Forsythe statistical test with a Games‐Howell correction, ****P value <0.0001. *(D)* Intracellular calcium signalling was measured in hiPSC‐ derived live myofibres using an Axio observed microscope at day 45 post‐differentiation. Different regions were tracked to record the fluorescent intensity at a 10× magnification during 30 s and an interval of 200.0 ms corresponding to 151 frames. Regions of interest (ROIs) corresponding to muscle fibres were selected and intensity of fluorescence was acquired using the Imaris software. *(E)* Scattergram displays the amplitude of calcium uptake and release in 50 fibres randomly selected in different fields for each sample (*y*‐axis). Data were analysed using a Kruskal–Wallis non‐parametric test (ns: non‐significant; ****P value <0.0001).

### Calcium homeostasis is altered in FSHD myotubes

As we have observed, a global down‐regulation of sarcomeric proteins required for calcium‐dependent force generation and sarcomere contraction, we analysed both the frequency of contraction and the amplitude of calcium entry and release in hiPSC‐derived innervated muscle fibres for two controls, two FSHD1, two FSHD2 by live imaging (*Figure* 5B–D, *Movie* S1). Of note, for FSHD1, we compared cells derived from a patient with mosaicism, that is, a clone carrying a short D4Z4 allele and its isogenic control with a long allele.

By measuring fibre shifting over time, we observed a marked and highly significant decrease in the length of contraction of individual muscle fibres with length of contraction in FSHD fibres being 61% to 36% of the length of contraction determined in control fibres (*P* value <0.0001, *Figure* 5C). These differences occur in all four FSHD samples, in particular when comparison was made between the healthy and diseased clone from a patient with mosaicism. In FSHD muscle fibres, this decreased fibre contraction correlates with a significant decrease in the amplitude of release and/or reuptake of Ca2+ (46% to 84% compared with healthy cells, *P* value <0.0001, *Figure* 5D,E). We concluded from these data in live cells of significant differences in calcium handling in FSHD cells with consequences on the strength of fibre contraction (length, peaks prominence, and interval between peaks), reminiscent of previous observations in demembranated isolated fibres^25^ but also mimicking the typical weakening considered as a cardinal feature of the disease.

## Discussion

A top priority in FSHD research is to identify the link between the genetic alteration and the muscle defect. Indeed, how this genetic alteration triggers the typical progressive weakening that precedes inflammation or fat infiltration secondary to the muscle atrophy remains largely unknown, including the place of DUX4 and the numerous DUX4 targets in this cascade. We previously showed that the pathogenic *DUX4‐fl* transcript as well as some of the DUX4 targets are produced at a comparable level in cells from FSHD1 or FSHD2 patients but also from patients affected with BAMS, a rare unrelated developmental syndrome, despite the absence of muscle manifestation that is specific to FSHD.^12^, ^13^ However, as pathways that associate *DUX4* expression to the FSHD muscle pathogenesis are not defined, identification of biomarkers of FSHD muscle function remain critical for understanding the process leading to the pathology but also for the definition of readouts to be used for drug design, outcome measures, and monitoring of therapies aimed at correcting the functional defect.

Network‐based approaches have been widely used for efficient identification of new diseases genes or reveal biological processes perturbed in diseases. With this perspective, we used MOGAMUN, a novel algorithm that integrates expression data with the simultaneous exploration of multiplex networks (pathways, protein–protein interactions, and co‐expression) to retrieve active modules specifically dysregulated in diseases.^15^ As compared with existing tools such as jActiveModules,^26^ COSINE,^27^ and PinnacleZ, limited to the analysis of single networks, MOGAMUN takes into account different biological sources of physical and/or functional interactions. It also optimizes both the differential expression score and the density of interactions as other tools retrieve either modules with 2–3 nodes or thousands of occurrences, limiting further interpretations.^15^

Compared with transcription analyses on muscle cells only,^10^, ^28^, ^29^ our analysis provides an additional layer of sophistication for identification of pathways associated with FSHD as our cells are under continuous mechanical and electrical stimulations mediated by motor neurons that co‐differentiate with muscle fibres and are known to influence the transcriptional response. By using pluripotent cells from patients displaying the diverse disease‐associated genotypes (FSHD1, FSHD2, and mosaicism) into functional and contractile innervated muscle fibres, we provide strong evidence that modifications in the expression of genes encoding proteins forming the sarcomere and contractile apparatus contribute to the disease phenotype. Differences between FSHD samples likely reflect the variability in gene expression between patients; a differential that is corrected by networks analysis that highlights in common between patient‐derived samples.

In FSHD cells, we observed enrichment in genes involved in cellular processes related to ECM, adhesion and migration, and a decreased in genes expressed in myogenic precursors such as *VCAM1* or *ERBB3* as reported.^23^, ^30^ In contrast to previous reports on cellular models with induced *DUX4* overexpression^31^ or mouse model,^23^ we did not find any enrichment in genes related to breast, lung, colon, thyroid cancer, gliomas and leukaemia, inflammation, apoptosis, or response to oxidative stress, most of which are not associated to FSHD clinical spectrum. We did not observe any specific involvement of MAP kinases signalling as compared with data obtained in *DUX4*‐induced models,^32^ consistent with the low level of *DUX4* and DUX4 target genes expression. Our analysis highlights the involvement of other pathways, relevant to muscle function and patient's phenotype, including extra muscular symptoms such as phosphatidyl inositol signalling required for Ca^2+^ release^33^ and the PI3K/AKT pathway that plays a critical role in myotube hypertrophy/atrophy, some of which were confirmed in muscle biopsies. Consistent with previous reports indicating the role of the WNT/β catenin signalling pathway in FSHD muscle phenotype but also retinal telangiectasia,^31^ we noticed a specific dysregulation of *WNT4* and associated SFRP proteins in FSHD. During development, the level of SFRPs is inverted compared to WNT4 along the dorsal to ventral axis.^34^ Consistently, we found an inverted level of expression between *WNT4* and of the four SFRP members acting as antagonists in FSHD cells compared with controls evoking a potential defect in cell signalling pathways during FSHD cells differentiation. In addition, decreased *MYOD* and increases *p73* expression suggests a delayed muscle differentiation associated to a decreased expression of sarcomeric proteins and proteins involved in muscle contraction in agreement with the functional defects evidenced in live cells. In FSHD cells, the implication of ERBB3 and PI3K/AKT pathways together with findings the role of PAX7 and PAX7 targets^29^ underlines a possible developmental origin of the disease, a plausible explanation given the muscle specificity and asymmetry.

In muscle, a significant amount of the contractile force is transmitted across the sarcolemma with an essential role for the ECM in transmitting the force and maintaining myofibre integrity.^35^ Loosened attachment and changes in ECM stiffness might thus predispose patients to muscle weakness and progressive muscle loss, reduced repair or regenerative capacity. In FSHD cells and tissues, decreased expression of genes encoding proteins of the TROPONIN complex, TROPOMYOSIN, TROPOMODULIN, ACTIN, TITIN, and NEBULIN, as also revealed by proteomics analysis^36^, ^37^ points towards a defect in thin filament organization, strength, and resistance to tension. We did not notice any enrichment in specific splice variants for transcripts encoding these different factors. This observation is also consistent with previous data that revealed defects in specific stages of myogenic differentiation in FSHD muscles^29^ and implicated the actin cytoskeleton signalling in the disease.^37^, ^38^

Electron microscopy or immunochemistry did not reveal any disease‐specific features or ultrastructural changes in FSHD biopsies.^39^, ^40^ Individual fibres myofibrillar structure is preserved with atrophic and hypertrophic fibres.^40^ In severely affected muscles, the presence of small angular fibres is evocative of regeneration, inflammation or fibrosis as a sign of the myopathic process.^39^ However, misalignment of the contractile apparatus with increased distance between the sarcolemma and the nearest myofibrils observed in biopsies^38^ appeared to be consistent with pathways retrieved via MOGAMUN. Our RNA‐Seq analysis did not evidence any alteration in the expression of genes required for calcium storage but variations in the expression of genes required for excitation and fibre contraction coupling or encoding several voltage‐dependent calcium channels. This decreased gene dosage might explain the overall absence of major ultrastructural abnormalities in FSHD muscle biopsies. Hence, changes in the regulation excitation–contraction coupling might be associated with the cumulative muscle weakness as observed overtime in patients together with the decline in muscle performance, maximal voluntary force and involuntary twitch response related to fatigability.^41^

Numerous FSHD disease models have been developed and major efforts have been deployed to understand the role of *DUX4* overexpression in the disease process.^10^, ^23^, ^32^ However, the activity of muscle function has been largely overlooked so far in all of them and all current readouts used for drug identification are mostly indirect.

Our ‘muscle in a dish’ approach reveals that alterations in sarcolemmal proteins and excitation–contraction coupling are likely involved in the typical and progressive weakening that characterizes FSHD clinical phenotype, in agreement with previous reports,^36^, ^38^, ^41^ including in isolated demembranated muscle fibres with FSHD fibres showing 70% of force compared to healthy donors.^25^ Decreased expression of sarcomeric proteins might also reveal delayed myogenic differentiation and myotube formation, as suggested in human embryonic stem cells‐derived muscle fibres,^21^ the link to *PAX7* target genes, considered as more robust marker compared to DUX4 targets^29^ or the implication of MyoD target genes,^36^ all relevant to the muscle phenotype. In FSHD, interventions that would restore the actin–myosin interface or regulate calcium entry might represent novel therapeutic options to reduce muscle weakness in FSHD and pave the way to therapies to improve muscle function and the life of patients.

## Conflict of interest

No conflict of interest declared.

## Funding

This study was funded by the ‘Association Française contre les Myopathies’ (AFM; TRIM‐RD) and *Fondation Maladies Rares*. C. L. was the recipient of a fellowship from the French Ministry of Education. M. D. is the recipient of a fellowship from the French Ministry of Education. The project leading to this publication has received funding from the Excellence Initiative of Aix‐Marseille University‐A*Midex, a French ‘investissement d'avenir programme’ AMX‐19‐IET‐007 through the Marseille Maladies Rares (MarMaRa) Institute (PhD fellowship to C. L. and K. M.).

## Supporting information


**Data S1.** Supporting information

**Table S1.** List of hiPSCS clones and biopsies. BAMS‐Case 1; BAMS‐Case 2 and BAMS‐Case 9 were described in [1]. All cells are described in [2]. GW: gestational week.

**Table S2.** Sequence of the primers used for RT‐qPCR. Primers for DUX4 and DUX4 targets were described in [3].
**Figure S1. RNA Seq analysis reveals different pattern of expression between FSHD and BAMS patients A.** Heatmap of RNAseq data (TPM values with a row sum > 1, distance: Manhattan, Clustering: Ward.D2) for gene expressed at D30 of differentiation in hiPSC‐derived innervated muscle fibres. Unsupervised hierarchical clustering separate BAMS from FSHD patients (FSHD2, FSHD1 and FSHD1‐mosaic). **B.** Volcano plots for genes differentially regulated in FSHD1, FSHD1 mosaic clone, FSHD2 or BAMS cells versus controls. Fold changes (FC log 2) are compared to the number of reads (logCounts). Black dots represent genes that did not reach significance whereas dysregulated genes are shown in red. **C.** Venn diagrams for genes upregulated in hiPS‐derived muscle fibres from the different categories of patients compared to controls. **D**. Venn diagrams for genes downregulated in hiPS‐derived muscle fibres from the different categories of patients compared to controls. **E**. Distribution of the different types of transcripts identified by RNA Seq. Orange, long non‐coding RNAs, Green, pseudogenes, Cyan, Protein coding RNAs. Purple, others.
**Figure S2. FSHD1 active modules of genes encoding extracellular matrix (ECM) components and sarcomeric proteins involved in muscle contraction.** Representative active modules sampled from the 23 accumulated Pareto font of 30 runs for FSHD1 datasets using the MOGAMUN algorithm. Integration of protein–protein interactions (blue lines), biological pathways (orange lines) and co‐expression data (yellow lines) with our lists of DEGs enabled the identification of active modules for each category of samples. Upregulated nodes are coloured in red and down‐regulated ones, in green. The intensity of the colour reflects the fold‐change. Thickness of the dark line around each rectangle reflects the level of significance (FDR < 0.05 and −1 < FC > 1). Each active module contains between 15 and 16 genes. Genes corresponding to each nodes were analysed using g:Profiler to define corresponding molecular function and p‐value. **A.** ECM organization, p‐value 8.6e‐19. **B.** ECM organization p‐value 9.9e‐11**. C.** ECM organization, p‐value 8.4e‐16. **D.** Actin‐Myosin filament sliding or muscle filament sliding, p‐value 7.3e‐39. **E.** Actin‐Myosin filament sliding or muscle filament sliding, p‐value 7.9e‐35. **F.** Actin‐Myosin filament sliding or muscle filament sliding, p‐value 7.3e‐39. **G.** Actin‐Myosin filament sliding or muscle filament sliding, p‐value 4.5 e‐8.
**Figure S3. FSHD1 active modules of genes involved in transmembrane receptor Tyrosine kinase signalling. A.** p‐value 4.2e‐14. **B.** p‐value 9.2e‐8. **C.** p‐value 8.16e‐ 17. **D.** p‐value 8.2e‐17. **E.** p‐value 2.64e‐14. **F.** p‐value 3.3e‐18. **G.** p‐value 3.3e‐18.
**Figure S4.** FSHD1 active modules of genes involved in Phosphatidyl Inositol mediated signalling. **A.** p‐value 4e‐16. **B.** p‐value 9.5e‐19. **C.** p‐value 2.13e‐14. **D.** p‐value 8.2e‐17.
**Figure S5. FSHD2 active modules. A.** Actin‐Myosin filament sliding or muscle filament sliding, p‐value 5.4e‐36**. B.** Actin‐Myosin filament sliding or muscle filament sliding, p‐value 3.16e‐29. **C.** Actin‐Myosin filament sliding or muscle filament sliding, p‐value 1.1.16e‐27. **D.** Nodes associated to positive regulation of macromolecule synthesis, p‐value 4.2e‐11. **E.** Nodes associated to regulation of transcription, p‐value 2.2e‐13. **F.** Regulation of transcription, p‐value 3e‐12. **G.** Node associated to mitotic cell cycle checkpoint, p‐value 9e‐10. **H.** Node associated to RNA splicing via splicesome, p‐value 4.3e‐16.
**Figure S6. BAMS active modules of genes encoding extracellular matrix (ECM) components. A.** p‐value 4.3e‐16. **B.** p‐value 2.15e‐16. **C.** p‐value 2.5e‐21. **D.** p‐value 3.6e‐ 17. **E.** p‐value 8.4e‐16.
**Figure S7. BAMS active modules of genes involved in transmembrane receptor Tyrosine kinase signalling. A.** p‐value 1.14e‐15**. B.** p‐value 1.85e‐13. **C.** p‐value 3.3e18. This node is also associated to ERBB2 signalling, p‐value 3.6e‐16. **D.** p‐value 8.16e‐17. This node is also associated to ERBB2 signalling, p‐value 1.8e‐16. **E.** p‐value 5.1e‐14; ERBB2 signalling, p‐value 8.5e‐13. **F.** p‐value 1.85e‐13. This node is also associated to Phosphatidyl inositol 3 kinase signalling, p‐value 1.5e‐12.
**Figure S8. Additional active module retrieved by MOGAMUN in BAMS muscle fibres. A.** Apoptotic process, p‐value 7.6e‐12. **B.** Cornification, p‐value 1.2e‐21. **C.** Skin development, p‐value 4.1e‐7.
**Figure S9. Nodes associated with DNA replication initiation in FSHD1, FSHD2 and BAMS muscle fibres. A.** FSHD1, p‐value 4.3e‐27. **B.** FSHD1, p‐value 4.3e‐27. **C.** p‐value 8.7e‐21. **D.** FSHD1, p‐value 4.3e‐27. **E.** FSHD2, p‐value 2.37e‐31. **F.** FSHD2, p‐value 8.9e‐ 29. **G.** BAMS, p‐value 8.9e‐18. **H.** BAMS, p‐value 2e‐25.
**Figure S10. Validation of DEG in additional hiPSC‐derived muscle fibres and FSHD biopsies. A.** RT‐qPCR was performed on biological and technical in triplicates for hiPSC‐derived muscle fibres for each group of samples. FSHD1 short correspond to a clone containing the contracted D4Z4 (3 RUs) from mosaic patient and FSHD1 long correspond to its isogenic control (15 RUs). Statistical significance was determined by Kruskal‐Wallis statistical test. **B**. RT‐qPCR was performed in technical triplicates for each biopsy sample. For each group, 2 different biopsies from 2 different individuals were used (Supplementary table 1). Expression was normalized to three housekeeping genes (*GAPDH*, *HPRT* and *PPIA*). Statistical significance was determined by Kruskal‐Wallis statistical test. * p‐value<0.05, ** p‐value<0.005, *** p‐value< 0.0005 and **** p‐value.<0.00005

**Movie S1.** Movie.
