# Supplementary material for: Facioscapulohumeral dystrophy weakened sarcomeric contractility is mimicked in induced pluripotent stem cells‐derived innervated muscle fibres
Source: J Cachexia Sarcopenia Muscle. 2021 Dec 3;13(1):621–35. doi: 10.1002/jcsm.12835 (PMC8818656; doi:10.1002/jcsm.12835)
Supplement: Supplementary file 1 — Data S1. Supporting information [file JCSM-13-621-s002.pdf]

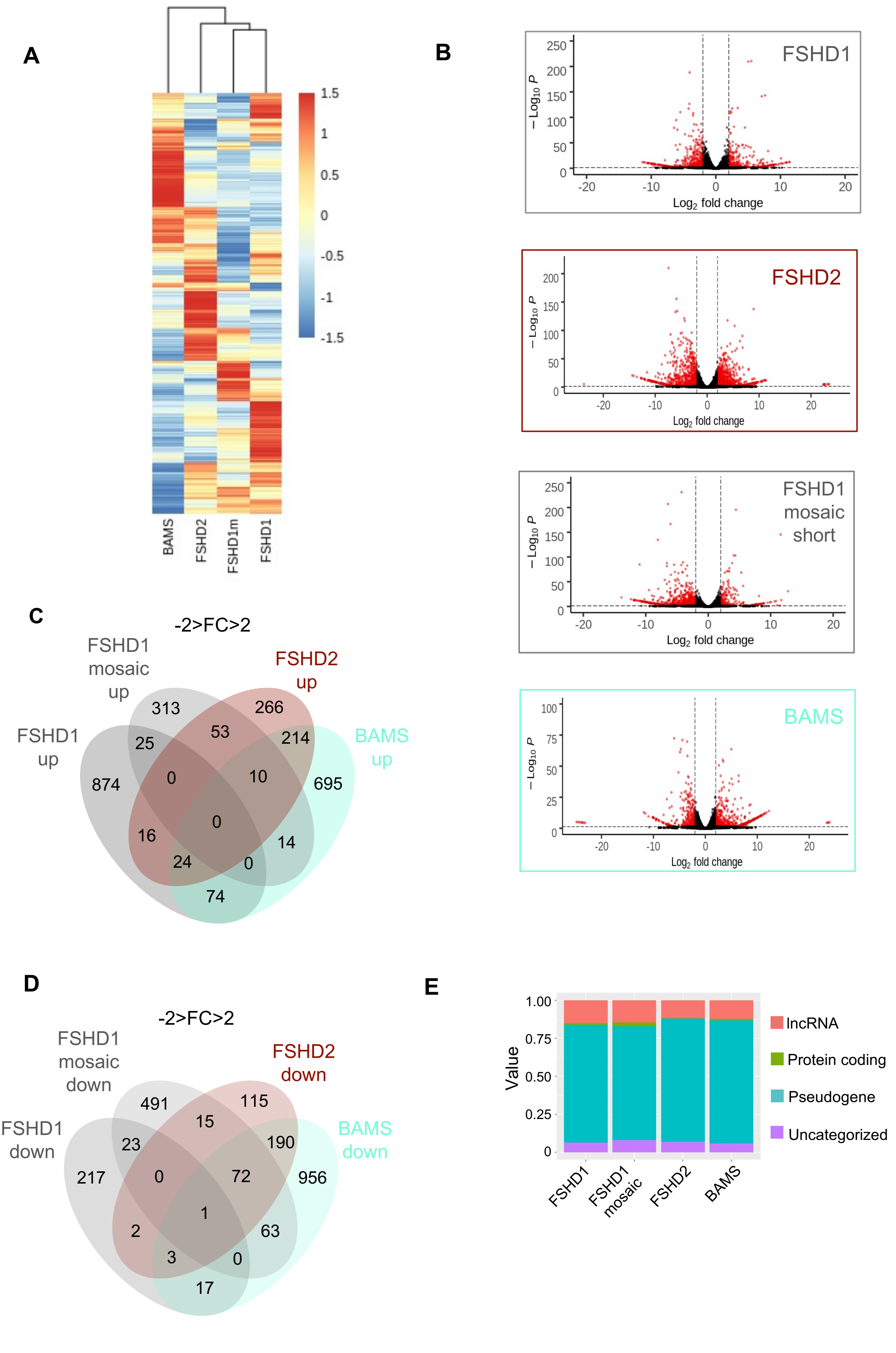
A
1.5
1
0.5
0
-0.5
-1
-1.5
BAMS
FSHD2
FSHD1m
FSHD1
B
FSHD1
FSHD2
FSHD1 mosaic short
BAMS
− Log10 P
Log2 fold change
C
-2>FC>2
FSHD1 up
FSHD1 mosaic up
FSHD2 up
BAMS up
874
313
25
266
53
0
214
10
695
0
16
24
0
14
74
D
-2>FC>2
FSHD1 down
FSHD1 mosaic down
FSHD2 down
BAMS down
217
491
23
115
15
0
190
72
956
1
2
3
0
63
17
E
Value
1.00
0.75
0.50
0.25
0
lncRNA
Protein coding
Pseudogene
Uncategorized
FSHD1
FSHD1 mosaic
FSHD2
BAMS

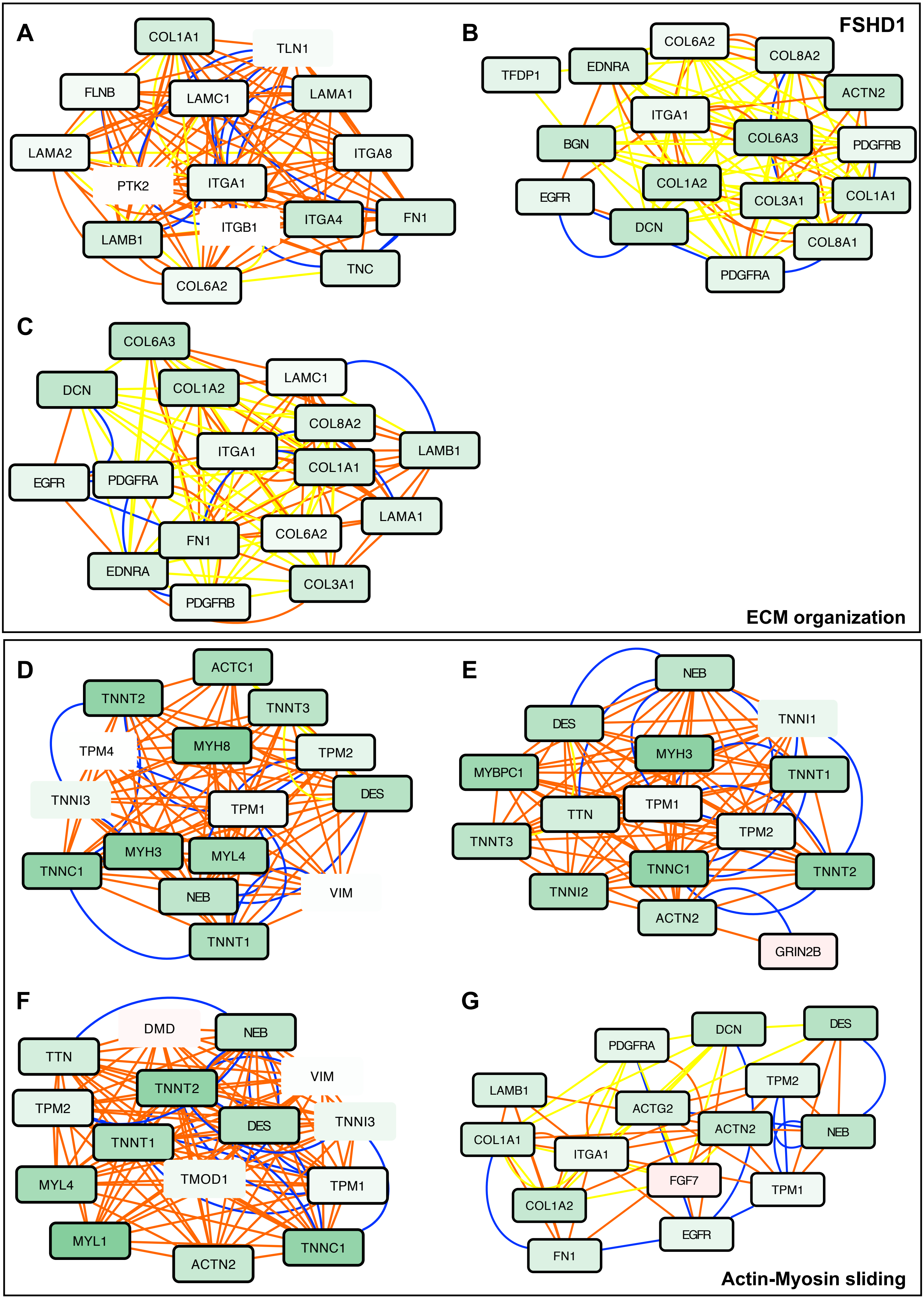

Laberthonnière *et al.* **Supplementary figure 2**

FSHD1
Tyrosine Kinase signalling

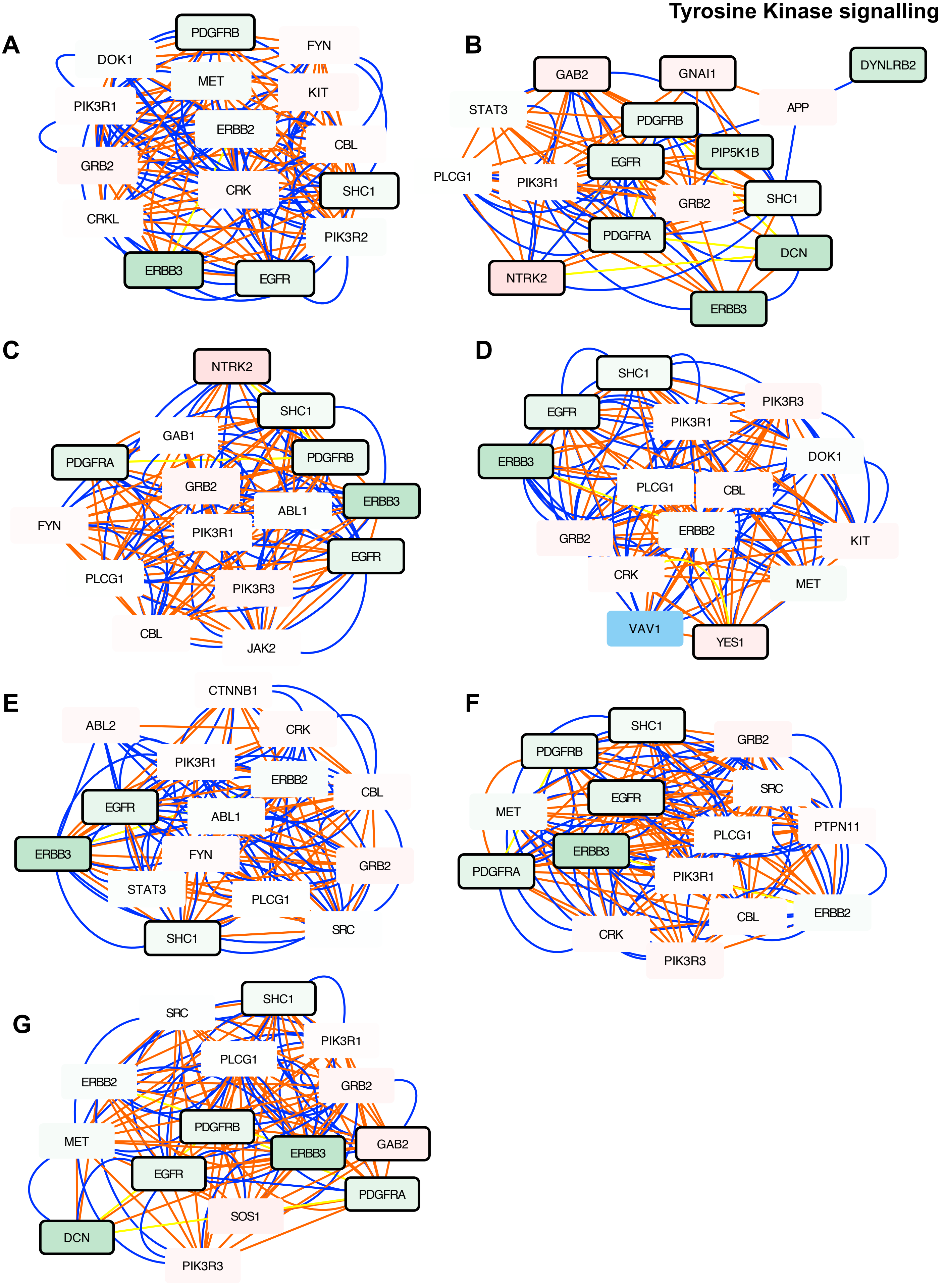

FSHD1
Phosphatidyl inositol signalling

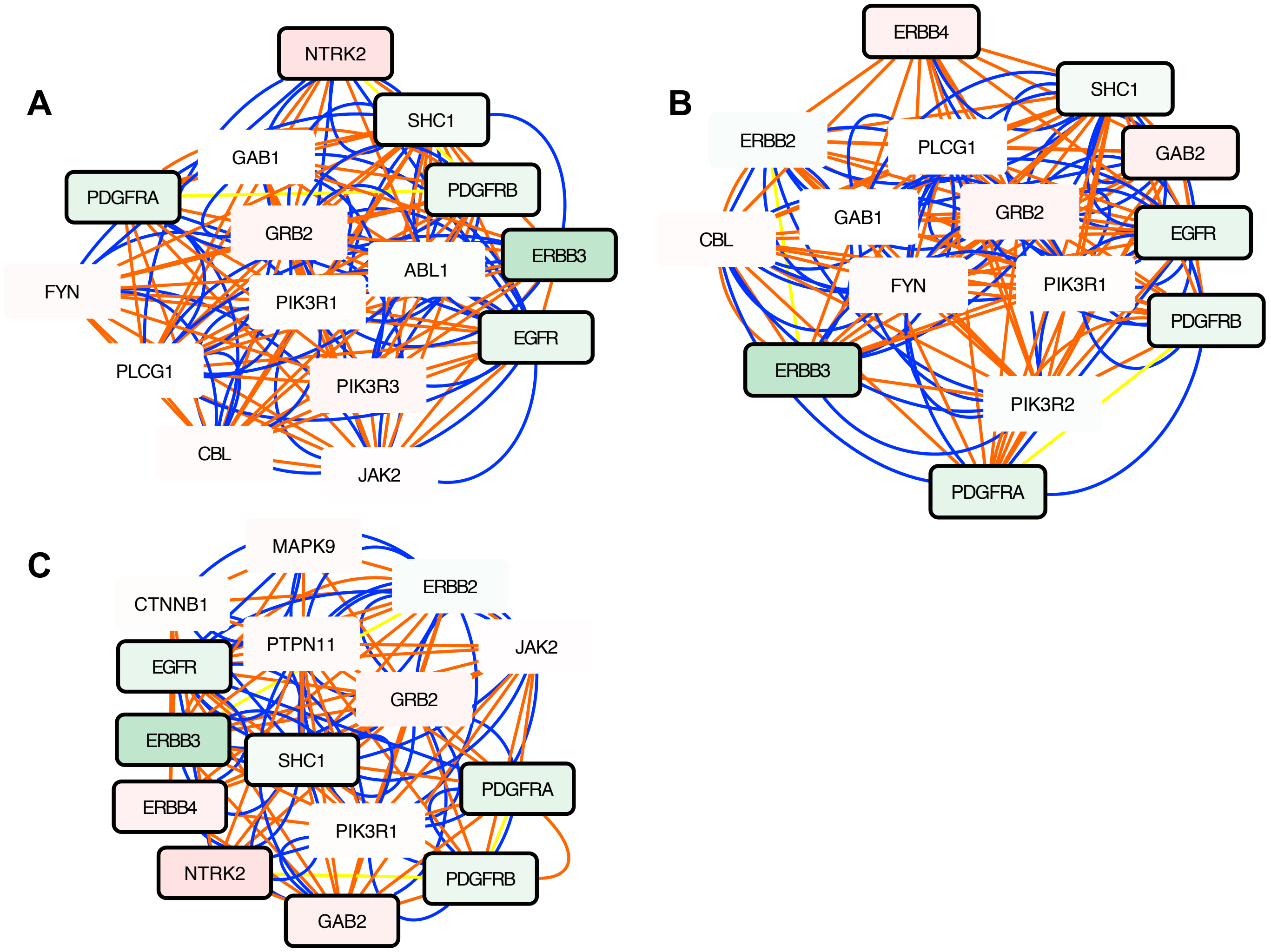

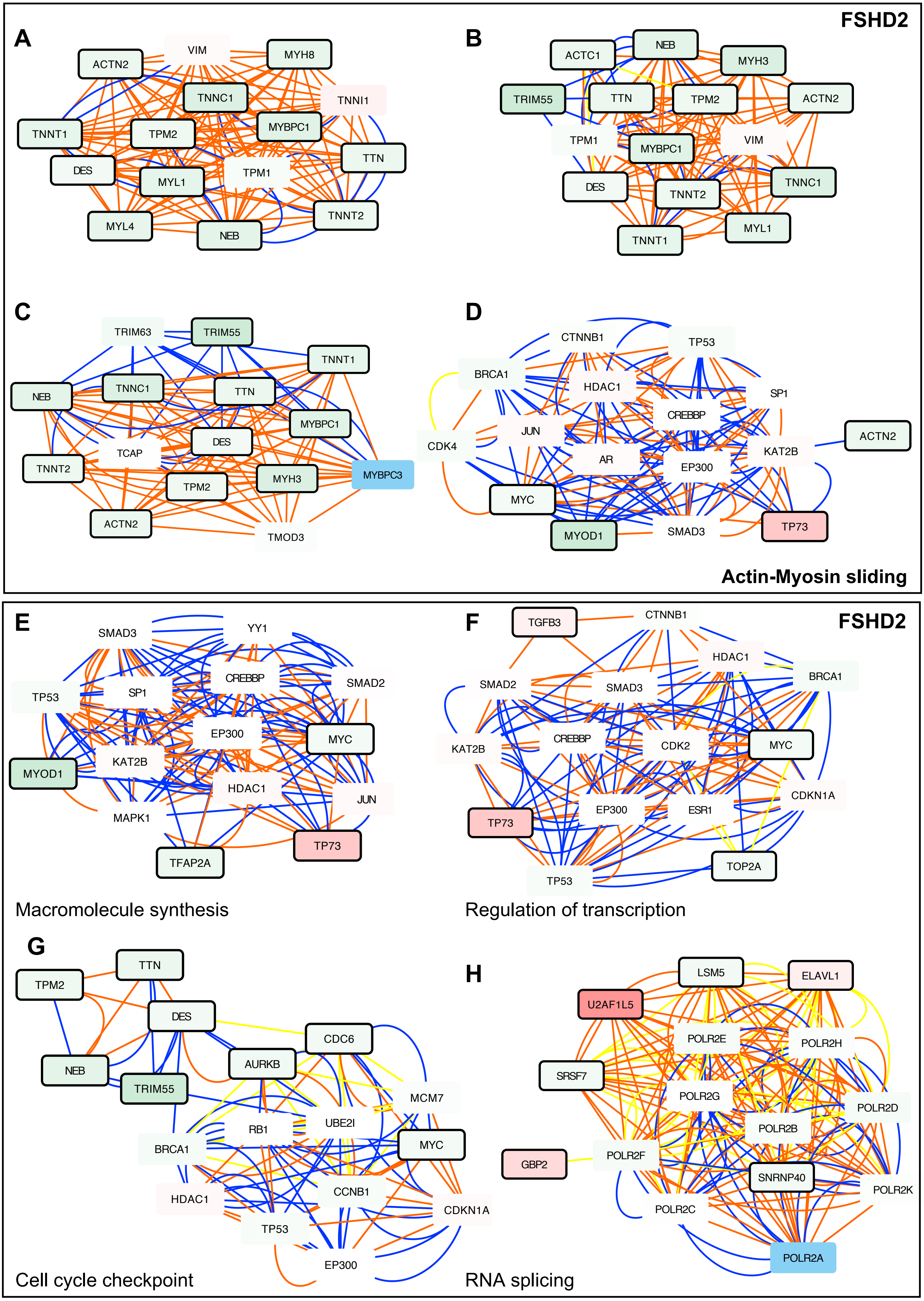
FSHD2
A
ACTN2
VIM
MYH8
TNNC1
TNNI1
TNNT1
TPM2
MYBPC1
TTN
DES
MYL1
TPM1
TNNT2
MYL4
NEB
B
ACTC1
NEB
MYH3
TRIM55
TTN
TPM2
ACTN2
TPM1
MYBPC1
VIM
DES
TNNT2
TNNC1
TNNT1
MYL1
C
TRIM63
TRIM55
TNNT1
NEB
TNNC1
TTN
MYBPC1
TCAP
DES
TNNT2
TPM2
MYH3
MYBPC3
ACTN2
TMOD3
D
CTNNB1
TP53
BRCA1
HDAC1
SP1
CREBBP
JUN
CDK4
ACTN2
AR
KAT2B
EP300
MYC
MYOD1
SMAD3
TP73
Actin-Myosin sliding
E
SMAD3
YY1
CREBBP
SMAD2
TP53
SP1
EP300
MYC
MYOD1
KAT2B
HDAC1
JUN
MAPK1
TP73
TFAP2A
Macromolecule synthesis
F
TGFB3
CTNNB1
FSHD2
HDAC1
BRCA1
SMAD2
SMAD3
CREBBP
KAT2B
CDK2
MYC
CDKN1A
EP300
ESR1
TP73
TOP2A
TP53
Regulation of transcription
G
TTN
TPM2
DES
CDC6
NEB
AURKB
TRIM55
MCM7
RB1
UBE2I
BRCA1
MYC
CCNB1
HDAC1
CDKN1A
TP53
EP300
Cell cycle checkpoint
H
LSM5
ELAVL1
U2AF1L5
POLR2E
POLR2H
SRSF7
POLR2G
POLR2D
POLR2B
GBP2
POLR2F
SNRNP40
POLR2K
POLR2C
POLR2A
RNA splicing

BAMS
ECM organization

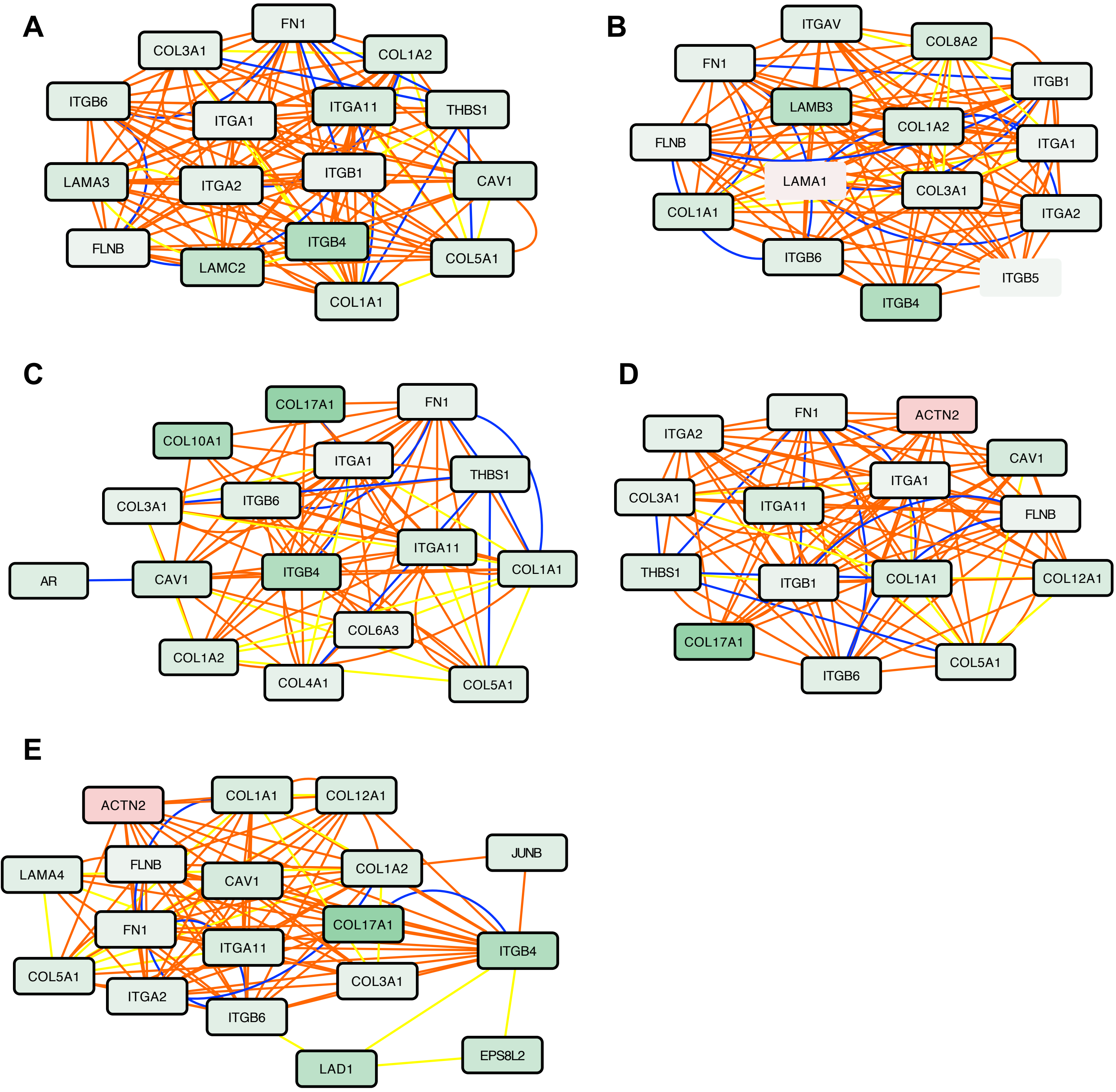

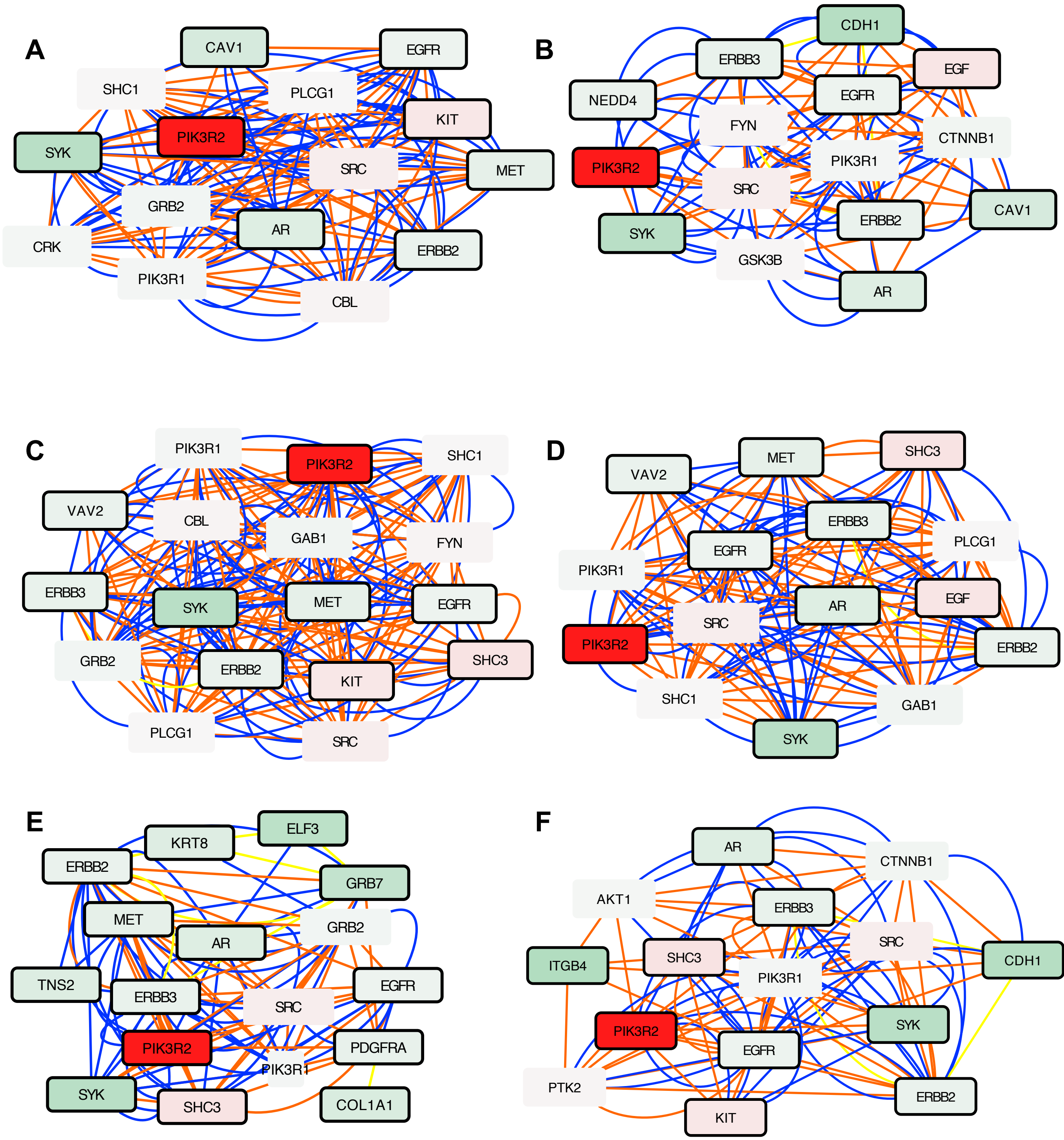
A
CAV1
EGFR
SHC1
PLCG1
KIT
PIK3R2
SYK
SRC
MET
GRB2
AR
CRK
ERBB2
PIK3R1
CBL
B
CDH1
ERBB3
EGF
NEDD4
EGFR
FYN
CTNNB1
PIK3R2
PIK3R1
SRC
CAV1
ERBB2
SYK
GSK3B
AR
C
PIK3R1
PIK3R2
SHC1
VAV2
CBL
GAB1
FYN
ERBB3
SYK
MET
EGFR
GRB2
ERBB2
KIT
SHC3
PLCG1
SRC
D
MET
SHC3
VAV2
ERBB3
PLCG1
EGFR
PIK3R1
AR
EGF
SRC
PIK3R2
ERBB2
SHC1
GAB1
SYK
E
ELF3
KRT8
ERBB2
GRB7
MET
GRB2
AR
TNS2
EGFR
ERBB3
SRC
PIK3R2
PDGFRA
PIK3R1
SYK
SHC3
COL1A1
F
AR
CTNNB1
AKT1
ERBB3
SRC
ITGB4
SHC3
CDH1
PIK3R1
PIK3R2
SYK
EGFR
PTK2
ERBB2
KIT

A

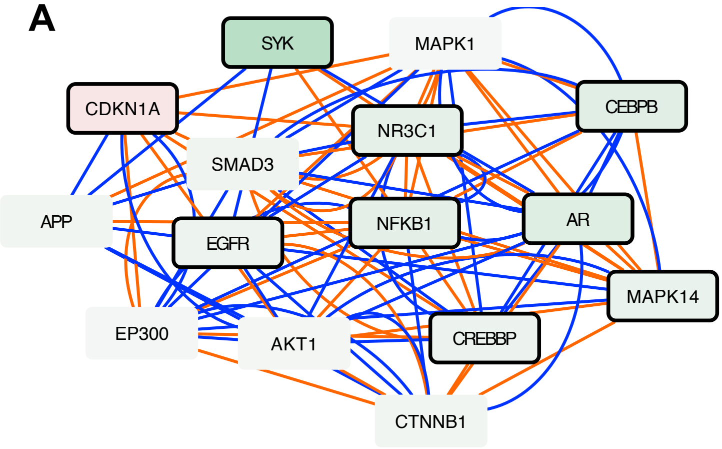

Apoptotic process

B

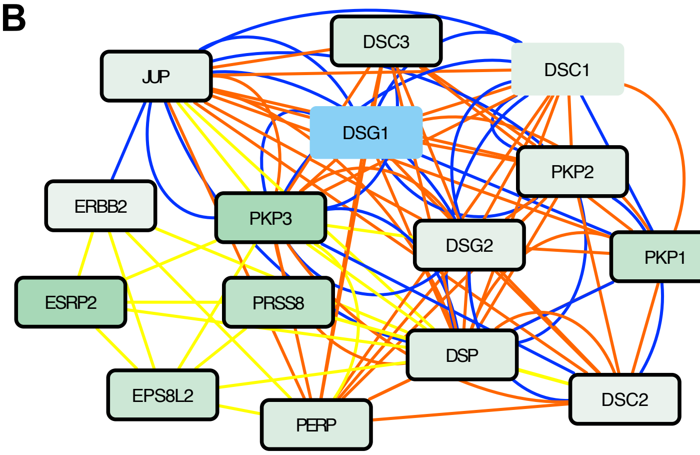

Cornificaton

C

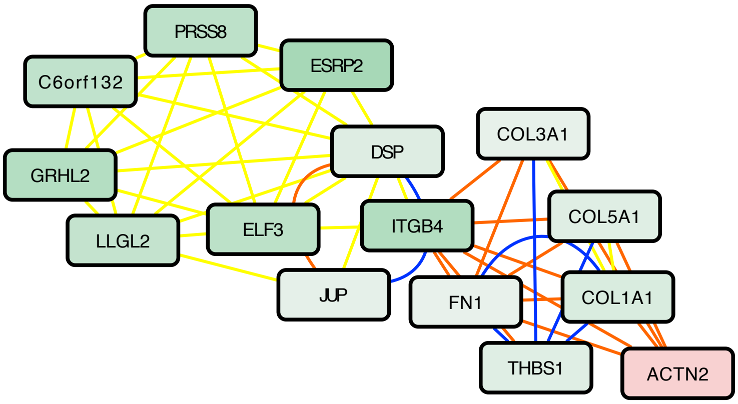

Skin development

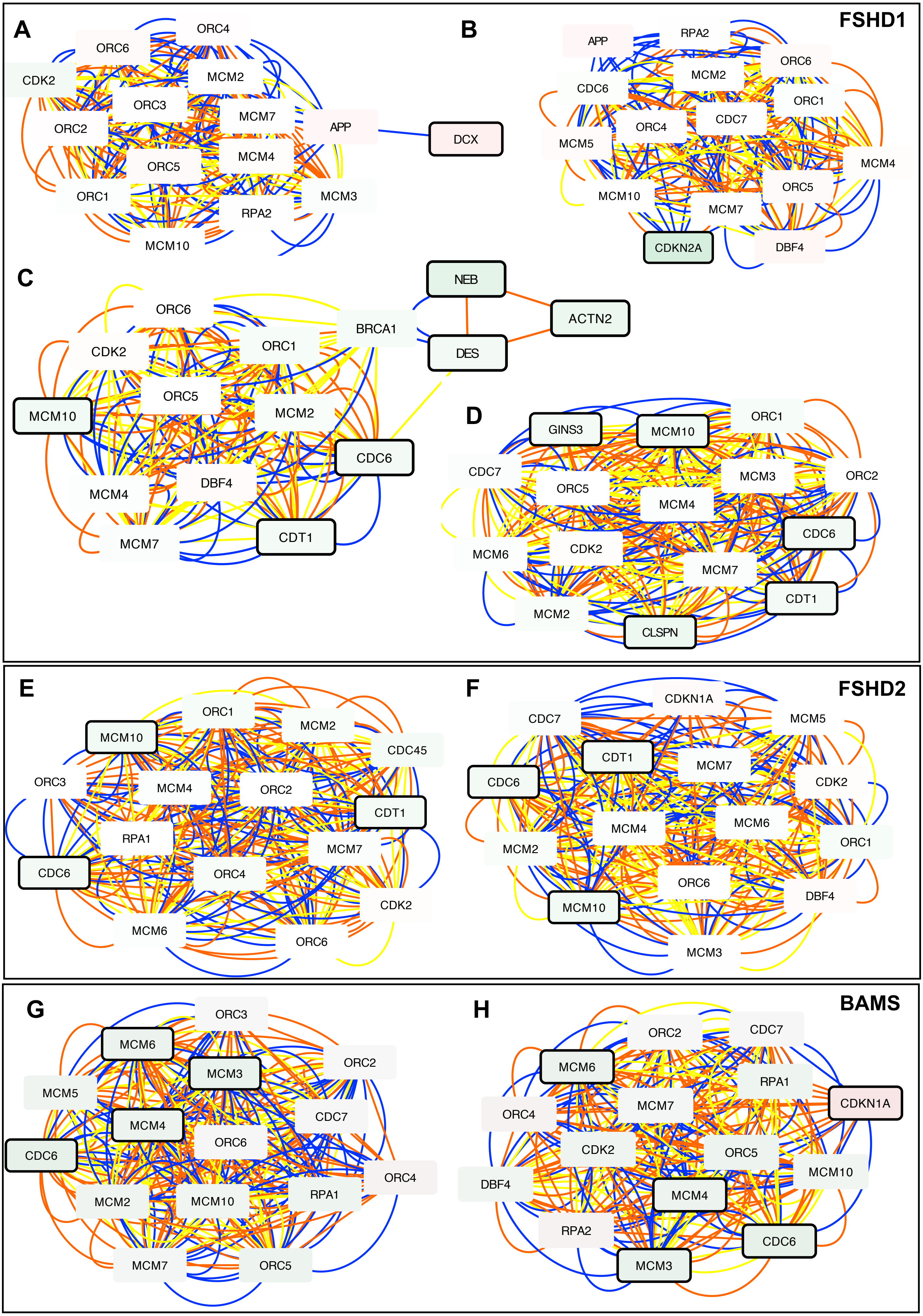
FSHD1
A
ORC4
ORC6
CDK2
MCM2
ORC3
MCM7
APP
DCX
ORC2
MCM4
ORC5
ORC1
MCM3
RPA2
MCM10
B
APP
RPA2
ORC6
MCM2
CDC6
ORC1
CDC7
ORC4
MCM5
MCM4
ORC5
MCM10
MCM7
CDKN2A
DBF4
C
NEB
ACTN2
DES
ORC6
BRCA1
CDK2
ORC1
ORC5
MCM10
MCM2
CDC6
DBF4
MCM4
CDT1
MCM7
D
GINS3
MCM10
ORC1
CDC7
ORC5
MCM3
ORC2
MCM4
CDC6
CDK2
MCM6
MCM7
CDT1
MCM2
CLSPN
E
ORC1
MCM10
MCM2
CDC45
ORC3
MCM4
ORC2
CDT1
RPA1
MCM7
CDC6
ORC4
CDK2
MCM6
ORC6
F
FSHD2
CDKN1A
CDC7
MCM5
CDT1
MCM7
CDC6
CDK2
MCM4
MCM6
MCM2
ORC1
ORC6
DBF4
MCM10
MCM3
G
ORC3
MCM6
MCM3
ORC2
MCM5
CDC7
MCM4
ORC6
CDC6
ORC4
MCM2
MCM10
RPA1
MCM7
ORC5
H
BAMS
ORC2
CDC7
MCM6
RPA1
MCM7
CDKN1A
ORC4
CDK2
ORC5
MCM10
DBF4
MCM4
RPA2
CDC6
MCM3

A

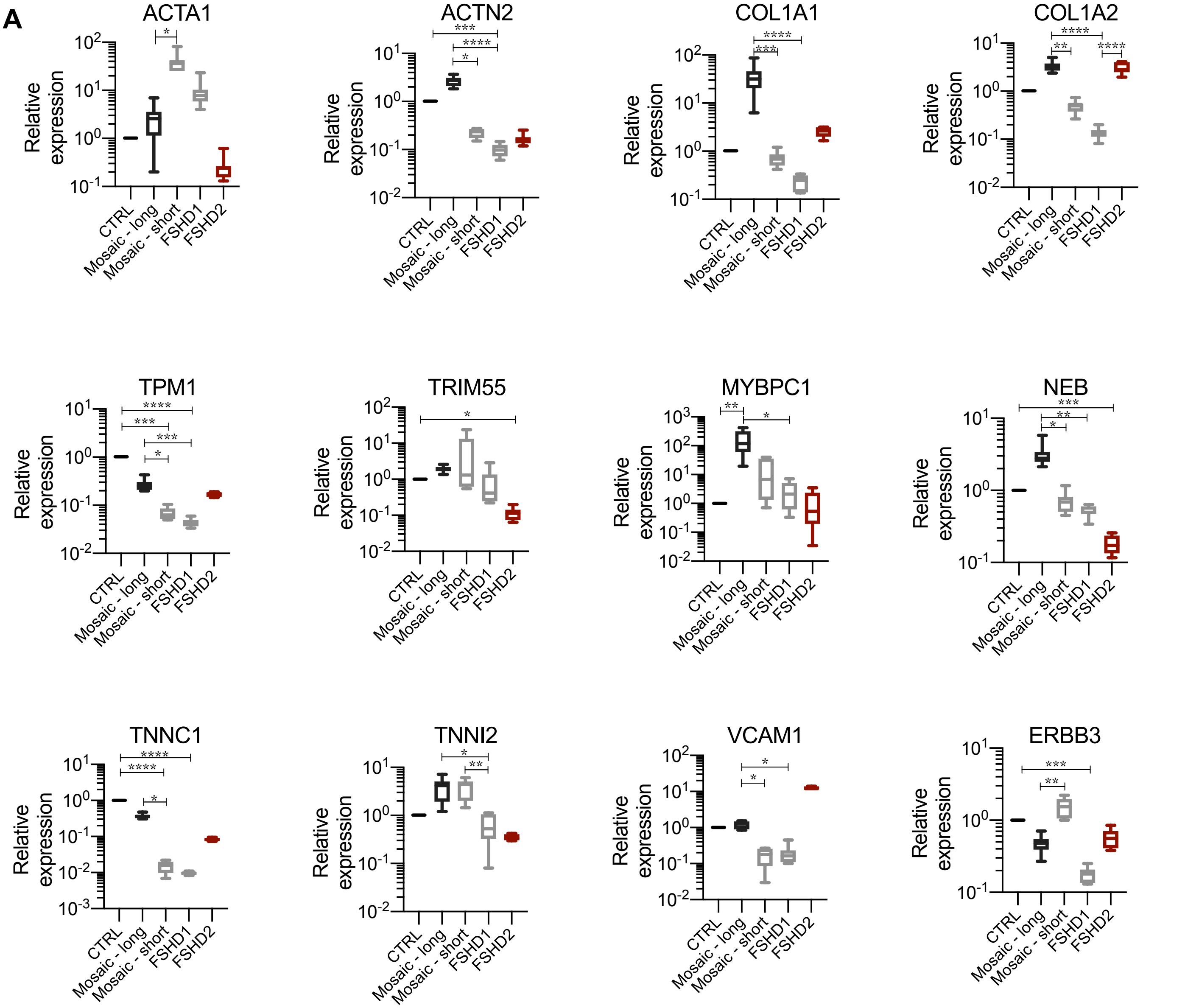
ACTA1
ACTN2
COL1A1
COL1A2
TPM1
TRIM55
MYBPC1
NEB
TNNC1
TNNI2
VCAM1
ERBB3
Relative expression
CTRL
Mosaic - long
Mosaic - short
FSHD1
FSHD2

B

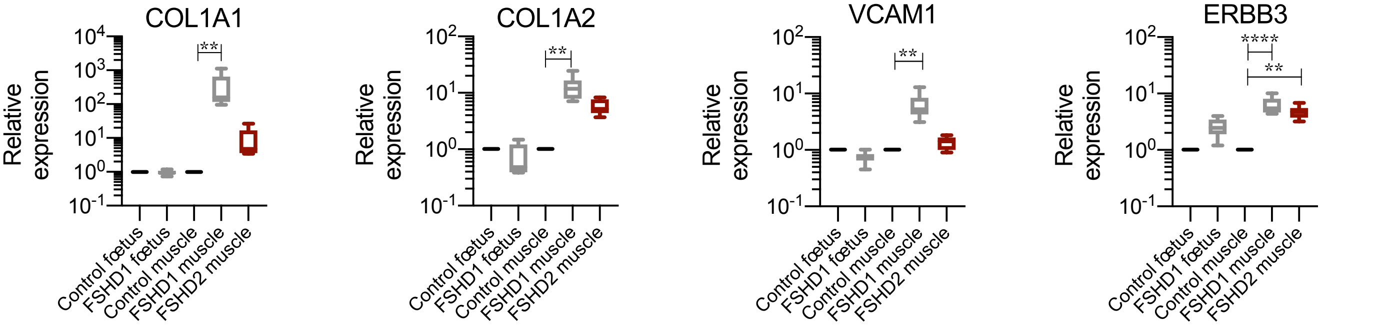
COL1A1
COL1A2
VCAM1
ERBB3
Relative expression
Control fœtus
FSHD1 fœtus
Control muscle
FSHD1 muscle
FSHD2 muscle
