# Supplementary material for: Facioscapulohumeral dystrophy weakened sarcomeric contractility is mimicked in induced pluripotent stem cells‐derived innervated muscle fibres
Source: J Cachexia Sarcopenia Muscle. 2021 Dec 3;13(1):621–35. doi: 10.1002/jcsm.12835 (PMC8818656; doi:10.1002/jcsm.12835)
Supplement: Supplementary file 3 — Table S2. Sequence of the primers used for RT‐qPCR. Primers for DUX4 and DUX4 targets were described in [3]. Figure S1. RNA Seq analysis reveals different pattern of expression between FSHD and BAMS patients A. Heatmap of RNAseq data (TPM values with a row sum > 1, distance: Manhattan, Clustering: Ward.D2) for gene expressed at D30 of differentiation in hiPSC‐derived innervated muscle fibres. Unsupervised hierarchical clustering separate BAMS from FSHD patients (FSHD2, FSHD1 and FSHD1‐mosaic). B. Volcano plots for genes differentially regulated in FSHD1, FSHD1 mosaic clone, FSHD2 or BAMS cells versus controls. Fold changes (FC log 2) are compared to the number of reads (logCounts). Black dots represent genes that did not reach significance whereas dysregulated genes are shown in red. C. Venn diagrams for genes upregulated in hiPS‐derived muscle fibres from the different categories of patients compared to controls. D. Venn diagrams for genes downregulated in hiPS‐derived muscle fibres from the different categories of patients compared to controls. E. Distribution of the different types of transcripts identified by RNA Seq. Orange, long non‐coding RNAs, Green, pseudogenes, Cyan, Protein coding RNAs. Purple, others. Figure S2. FSHD1 active modules of genes encoding extracellular matrix (ECM) components and sarcomeric proteins involved in muscle contraction. Representative active modules sampled from the 23 accumulated Pareto font of 30 runs for FSHD1 datasets using the MOGAMUN algorithm. Integration of protein–protein interactions (blue lines), biological pathways (orange lines) and co‐expression data (yellow lines) with our lists of DEGs enabled the identification of active modules for each category of samples. Upregulated nodes are coloured in red and down‐regulated ones, in green. The intensity of the colour reflects the fold‐change. Thickness of the dark line around each rectangle reflects the level of significance (FDR < 0.05 and −1 < FC > 1). Each active module c [file JCSM-13-621-s003.pdf]

Supplementary information

Supplementary table 1: List of hiPSCS clones and biopsies.

BAMS-Case 1; BAMS-Case 2 and BAMS-Case 9 were described in [1]. All cells are described in [2]. GW: gestational week.

	Diagnosis	Genotype	*SMCHD1* status	Age	Gender	iPS	Biopsies
AG08498	Healthy	>10	No mutation	1	Male	✓	
F-CT2	CTRL	>11	No mutation	13 GW	Male	▪	Deltoid
F-CT11	CTRL	>11	No mutation	22 GW	Male	▪	Quadriceps
A-CT3	CTRL	>11	No mutation	69	Female	▪	Quadriceps
A-CT2	CTRL	>11	No mutation	71	Male	▪	Quadriceps
12759	FSHD1	7RU	No mutation	51	Female	✓	
17706	FSHD1 Mosaic	2RU -25%	No mutation	56	Female	✓	
FSHD1-7UR-6	FSHD1	7RU	No mutation	26 GW	Female	▪	Quadriceps
FSHD1-2UR-1	FSHD1	2RU	No mutation	16 GW	Male	▪	Deltoid
FSHD1-2	FSHD1	N.A.	No mutation	66	Male	▪	Quadriceps
FSHD1-1	FSHD1	N.A.	No mutation	54	Male	▪	Quadriceps
14586	FSHD2	>10	c.573A>C; p.Q193P	67	Male	✓	
120521C	FSHD2	>10	p.L1031L	10	Female	✓	
11440	FSHD2	>10	c.2338+4A>G; p.S754*	37	Male	✓	Quadriceps
VENPI	FSHD2	N.A.	N.A.	45	Male	▪	Quadriceps
BAMS-1	BAMS	N/A	c.407A>G p.E136G	5	Male	✓	
BAMS-2	BAMS	N/A	c.403A>T p.S135C	28	Female	✓	
BAMS-9	BAMS	N/A	c.1259A>T p.D420V	3	Male	✓	

Supplementary table 2: Sequence of the primers used for RT-qPCR.

Primers for DUX4 and DUX4 targets were described in [3].

Gene	Forward Primer	Reverse Primer
DUX4-92	CAAGGGGTGCTTGCGCCACCCAC GT	
DUX4-116		GGGGTGCGCACTGCGCGCAGGT
MDB3L2	CAGGAGTGGGGTCAGCAGGAGGA	TTTGAGCTTCCCCAGAACAGGCAGG
ZSCAN4	TGCCTCCTGGATTCAAACA	TGTTCTATACCATCACTGGTCCTG
TRIM 43	ACCCATCACTGGACTGGTGT	CACATCCTCAAAGAGCCTGA
MBDL3	CGTTCACCTCTTTTCCAAGC	AGTCTCATGGGGAGAGCAGA
LEUTX	CCATCACTAGGGCCAGCAAA	CAGGATGAAACCCTCGCAGA
HPRT	TGATAGATCCATTCCTATGACTGTA GA	CAAGACATTCTTTCCAGTTAAAGTTG
GAPDH	AGCCACATCGCTCAGACAC	GCCCAATACGACCAAATCC
PPIA	ATGCTGGACCCAACACAAAT	TCTTTCACTTTGCCAAACACC
ZSCAN4	TGCCTCCTGGATTCAAACA	TGTTCTATACCATCACTGGTCCTG
ACTA1	TCCGTTGCTGCCATCGTAAA	TCCCGCCCCAAGCAAATAAA
ACTN2	GAGGGCAAGATGGTGTCGGATA	CTTCTCAGCCAGGTGTTCCAAG
COL1A1	GGACACAGAGGTTTCAGTGGT	GCACCATCATTTCCACGAGC
COL1A2	TGGTGAAGTGGGTCTTCCA	ACCCTTGGCACCAGTAAGG
TRIM55	AAGGTCTGGGGCAAATAGGG	AGCGCTAGGCAGATTACTAAC
TPM1	CTTAGAGCCAGGCACACACT	GCAGCCAAACACTAACCCTC
MYBPC1	TACCCCTGGACAACCAGTCT	TCAAAGCAGCAGAGTGCAGA
NEB	CACCTGGTAGTCGATGAGCC	TCTGGGTTTGGCCTTGTTGT
TNNC1	GGCCGCATCGACTATGATGA	CAGGACTCAGCTGGAGTTGG
TNNI2	GCCCTGCTGCCCAGATTCTA	CTCTTCAGCCGCATCGATCT
ERBB3	GAGGGACCCAGGTCTACGAT	TCACGATGTCCCTCCAGTCA
VCAM1	CACTGAATGGGAAGGTGACG	ACACTTGACTGTGATCGGCT

Legend to the supplementary figures

Supplementary figure 1. RNA Seq analysis reveals different pattern of expression between FSHD and BAMS patients

A. Heatmap of RNAseq data (TPM values with a row sum > 1, distance: Manhattan, Clustering: Ward.D2) for gene expressed at D30 of differentiation in hiPSC-derived innervated muscle fibers. Unsupervised hierarchical clustering separate BAMS from FSHD patients (FSHD2, FSHD1 and FSHD1-mosaic). **B.** Volcano plots for genes differentially regulated in FSHD1, FSHD1 mosaic clone, FSHD2 or BAMS cells versus controls. Fold changes (FC log 2) are compared to the number of reads (logCounts). Black dots represent genes that did not reach significance whereas dysregulated genes are shown in red. **C.** Venn diagrams for genes upregulated in hiPS-derived muscle fibers from the different categories of patients compared to controls. **D**. Venn diagrams for genes downregulated in hiPS-derived muscle fibers from the different categories of patients compared to controls. **E**. Distribution of the different types of transcripts identified by RNA Seq. Orange, long non-coding RNAs, Green, pseudogenes, Cyan, Protein coding RNAs. Purple, others.

Supplementary figure 2. FSHD1 active modules of genes encoding extracellular matrix (ECM) components and sarcomeric proteins involved in muscle contraction. Representative active modules sampled from the 23 accumulated Pareto font of 30 runs for FSHD1 datasets using the MOGAMUN algorithm. Integration of protein-protein interactions (blue lines), biological pathways (orange lines) and co-expression data (yellow lines) with our lists of DEGs enabled the identification of active modules for each category of samples. Up-regulated nodes are colored in red and down-regulated ones, in green. The intensity of the color reflects the fold-change. Thickness of the dark line around each rectangle reflects the level of significance (FDR<0.05 and -1<FC>1)**.** Each active module contains between 15 and 16 genes. Genes corresponding to each nodes were analyzed using g:Profiler to define corresponding molecular function and p-value. **A.** ECM organization, p-value $8.6e^{-19}$. **B.** ECM

organization, p-value $9.9e^{-11}$**.** **C.** ECM organization, p-value $8.4e^{-16}$. **D.** Actin-Myosin filament sliding or muscle filament sliding, p-value $7.3e^{-39}$. **E.** Actin-Myosin filament sliding or muscle filament sliding, p-value $7.9e^{-35}$. **F.** Actin-Myosin filament sliding or muscle filament sliding, p-value $7.3e^{-39}$. **G.** Actin-Myosin filament sliding or muscle filament sliding, p-value $4.5e^{-8}$.

Supplementary figure 3. FSHD1 active modules of genes involved in transmembrane receptor Tyrosine kinase signaling. A. p-value $4.2e^{-14}$**. B.** p-value $9.2e^{-8}$. **C.** p-value $8.16e^{-17}$. **D.** p-value $8.2e^{-17}$. **E.** p-value $2.64e^{-14}$**. F.** p-value $3.3e^{-18}$**. G.** p-value $3.3e^{-18}$.

Supplementary figure 4. FSHD1 active modules of genes involved in Phosphatidyl Inositol mediated signaling. A. p-value $4e^{-16}$**. B.** p-value $9.5e^{-19}$. **C.** p-value $2.13e^{-14}$. **D.** p-value $8.2e^{-17}$.

Supplementary figure 5. FSHD2 active modules. A. Actin-Myosin filament sliding or muscle filament sliding, p-value $5.4e^{-36}$**. B.** Actin-Myosin filament sliding or muscle filament sliding, p-value $3.16e^{-29}$. **C.** Actin-Myosin filament sliding or muscle filament sliding, p-value $1.1.16e^{-27}$. **D.** Nodes associated to positive regulation of macromolecule synthesis, p-value 4.2e-11. **E.** Nodes associated to regulation of transcription, p-value 2.2e-13. **F.** Regulation of transcription, p-value 3e-12. **G.** Node associated to mitotic cell cycle checkpoint, p-value $9e^{-10}$. **H.** Node associated to RNA splicing via splicesome, p-value $4.3e^{-16}$.

Supplementary figure 6. BAMS active modules of genes encoding extracellular matrix (ECM) components. A. p-value $4.3e^{-16}$. **B.** p-value $2.15e^{-16}$. **C.** p-value $2.5e^{-21}$. **D.** p-value $3.6e^{-17}$. **E.** p-value $8.4e^{-16}$.

Supplementary figure 7. BAMS active modules of genes involved in transmembrane receptor Tyrosine kinase signaling. A. p-value $1.14e^{-15}$**. B.** p-value 1.85^{e-13}. **C.** p-value 3.3e⁻

^{18}. This node is also associated to ERBB2 signaling, p-value $3.6e^{-16}$. **D.** p-value 8.16^{e-17}. This node is also associated to ERBB2 signaling, p-value $1.8e^{-16}$. **E.** p-value 5.1^{e-14}; ERBB2 signaling, p-value 8.5^{e-13}. **F.** p-value 1.85^{e-13}. This node is also associated to Phosphatidyl inositol 3 kinase signaling, p-value 1.5^{e-12}.

Supplementary figure 8. Additional active module retrieved by MOGAMUN in BAMS muscle fibers. A. Apoptotic process, p-value 7.6^{e-12}. **B.** Cornification, p-value 1.2^{e-21}. **C.** Skin development, p-value 4.1^{e-7}.

Supplementary figure 9. Nodes associated with DNA replication initiation in FSHD1, FSHD2 and BAMS muscle fibers. A. FSHD1, p-value 4.3^{e-27}. **B.** FSHD1, p-value 4.3^{e-27}. **C.** p-value 8.7^{e-21}. **D.** FSHD1, p-value 4.3^{e-27}. **E.** FSHD2, p-value 2.37^{e-31}. **F.** FSHD2, p-value 8.9^{e-29}. **G.** BAMS, p-value 8.9^{e-18}. **H.** BAMS, p-value 2^{e-25}.

Supplementary figure 10. Validation of DEG in additional hiPSC-derived muscle fibers and FSHD biopsies.

A. RT-qPCR was performed on biological and technical in triplicates for hiPSC-derived muscle fibers for each group of samples. FSHD1 short correspond to a clone containing the contracted D4Z4 (3 RUs) from mosaic patient and FSHD1 long correspond to its isogenic control (15 RUs). Statistical significance was determined by Kruskal-Wallis statistical test. **B**. RT-qPCR was performed in technical triplicates for each biopsy sample. For each group, 2 different biopsies from 2 different individuals were used (Supplementary table 1). Expression was normalized to three housekeeping genes (*GAPDH*, *HPRT* and *PPIA*). Statistical significance was determined by Kruskal-Wallis statistical test. * p-value<0.05, ** p-value<0.005, *** p-value<0.0005 and **** p-value<0.00005

Supplementary references

1. Gordon CT, Xue S, Yigit G, Filali H, Chen K, Rosin N, et al. De novo mutations in SMCHD1 cause Bosma arhinia microphthalmia syndrome and abrogate nasal development. Nat Genet. 2017;49:249-55. doi:10.1038/ng.3765
2. Dion C, Roche S, Laberthonniere C, Broucqsault N, Mariot V, Xue S, et al. SMCHD1 is involved in de novo methylation of the DUX4-encoding D4Z4 macrosatellite. Nucleic Acids Res. 2019;47:2822-39. doi:10.1093/nar/gkz005
3. Snider L, Geng LN, Lemmers RJ, Kyba M, Ware CB, Nelson AM, et al. Facioscapulohumeral dystrophy: incomplete suppression of a retrotransposed gene. PLoS Genet. 2010;6:e1001181. doi:10.1371/journal.pgen.1001181
